# Putrescine Mitigates the Biomass–β-Carotene Conflict in *Dunaliella salina* Under Thermal Stress

**DOI:** 10.3390/life15121807

**Published:** 2025-11-25

**Authors:** Jianxin Tang, Fantao Kong, Zhanyou Chi

**Affiliations:** MOE Key Laboratory of Bio-Intelligent Manufacturing, School of Bioengineering, Dalian University of Technology, Dalian 116024, China; tjx18809865913@mail.dlut.edu.cn (J.T.); kongfantao@dlut.edu.cn (F.K.)

**Keywords:** β-carotene biosynthesis, thermal stress, putrescine, biomass–carotenoid trade-off, NO signaling, temperature-dependent regulation

## Abstract

Heat-induced β-carotene synthesis in *Dunaliella salina* typically compromises biomass accumulation, resulting in a biomass–β-carotene trade-off. This study demonstrates that exogenous putrescine (Put) alleviates this conflict through temperature-dependent mechanisms. At 28 °C (optimal for growth), 10^−6^ M Put increased biomass by 9.52% and β-carotene yield by 10.72%, probably by accelerating electron transport and relatively mitigating the loss of photosynthetic function. At 34 °C (optimal for β-carotene synthesis), 10^−7^ M Put enhanced biomass by 9.68% and β-carotene yield by 35.71% through a process associated with nitric oxide (NO) accumulation, involving antioxidant synergy and controlled reactive oxygen species (ROS) signaling, which activated photoprotective carotenogenesis. At 40 °C (extreme thermal stress), 10^−7^ M Put maintained β-carotene levels 44.99% above the control despite a 2.50% biomass reduction, reflecting a shift toward photoprotection via elevated non-photochemical quenching (NPQ) and sustained electron transport beyond photosystem II (δ_RO_). Put’s hierarchical modulation of redox homeostasis, photosystem plasticity, and NO signaling underpinned its temperature-dependent efficacy. Peak NO levels correlated with β-carotene yield, while thermodynamic enzyme denaturation at 40 °C limited protection. These findings establish a temperature–concentration framework for Put application that alleviates the biomass–β-carotene trade-off under climate variability.

## 1. Introduction

β-carotene, an essential carotenoid and provitamin A, is widely utilized as a natural food colorant and dietary supplement [1,2]. The halotolerant green alga *Dunaliella salina* accounts for over 90% of global natural β-carotene production and can accumulate up to 14% of its dry biomass under stress conditions [3,4,5,6]. Among various induction strategies, heat stress effectively stimulates β-carotene biosynthesis but simultaneously inhibits biomass accumulation [7], resulting in a typical biomass–β-carotene trade-off that significantly restricts production efficiency and commercial scalability.

To overcome this limitation, plant growth regulators (PGRs) such as 24-epibrassinolide [8], fulvic acid [9], gibberellic acid [10], melatonin [11], and diethyl amino ethyl hexanoate [12] have been applied to improve stress tolerance and promote secondary metabolite accumulation in microalgae. However, chemical regulation strategies specifically targeting thermal stress in *Dunaliella salina* remain underexplored. Furthermore, the high cost of conventional PGRs hinders their large-scale industrial application, highlighting the need for cost-effective alternatives.

Polyamines (PAs), particularly putrescine (Put), are low-molecular-weight aliphatic amines that act as signaling molecules in both plant and microalgae responses to abiotic stress [13,14,15]. In higher plants, exogenous Put application has been demonstrated to mitigate heat stress in various species, such as tomato [16] and rice [17], by enhancing antioxidant defenses and protecting photosynthetic function. Similarly, in microalgae, Put has been shown to promote astaxanthin accumulation in *Haematococcus pluvialis* [15] and lipid accumulation in *Tetradesmus obliquus* [18] under stress conditions. Due to high membrane permeability, Put can scavenge ROS, stabilize photosynthetic membranes, and maintain redox homeostasis under stress conditions [19,20]. Furthermore, as a low-cost synthetic compound readily available commercially, Put represents a promising and economically viable alternative to conventional PGRs for large-scale microalgal cultivation [15]. In higher plants, Put has been shown to chelate Fe^2+^ [21], inhibit NADPH oxidase activity [22], activate antioxidant enzymes [23], and protect photosystems [24,25]. However, the temperature- and dose-dependent effects of Put on microalgae, which are fundamental for its application under realistic and fluctuating cultivation conditions, remain largely unexplored. Moreover, few studies have integrated redox signaling, photochemical performance, antioxidative responses, and the biomass–metabolite trade-off into a unified mechanistic framework.

Therefore, this study was designed to systematically investigate the potential and mechanism of exogenous Put in mitigating the biomass–β-carotene trade-off in *Dunaliella salina* under three thermal regimes: 28 °C (optimal for biomass accumulation), 34 °C (optimal for β-carotene synthesis), and 40 °C (extreme thermal stress). Specifically, biomass accumulation, β-carotene yield, pigment dynamics, photosynthetic performance, antioxidant responses, and NO signaling were evaluated. This comprehensive approach aims to fill the critical knowledge gap regarding Put’s function in microalgal thermotolerance and provide a theoretical basis for its application in optimizing β-carotene production.

## 2. Materials and Methods

### 2.1. Microalgal Strain and Culture Conditions

The microalga *Dunaliella salina* (CCAP 19/18) was obtained from the Culture Collection of Algae and Protozoa (Windermere, UK). In this study, the strain was inoculated into the medium according to the method described by Xi et al. (2022) [26]. The stock cultures were kept at 25 °C with continuous illumination at a light intensity of 40 μmol photons m^−2^ s^−1^, and agitated on an orbital shaker at 110 rpm. The initial pH was adjusted to approximately 8.5 ± 0.5. Semi-continuous cultivation was applied to keep the microalgae in the exponential growth phase, with cell densities maintained between 20 × 10^4^ and 100 × 10^4^ cells mL^−1^. Cells in the logarithmic growth phase were harvested by centrifugation at 3000× *g* for 2 min, and then resuspended in fresh medium for subsequent experiments.

### 2.2. Experimental Design and Put Treatment

To evaluate the impact of exogenous Put on biomass and β-carotene production in *Dunaliella salina*, cultures were subjected to three temperature conditions: 28 °C (optimal for growth), 34 °C (optimal for β-carotene synthesis), and 40 °C (extreme thermal stress), as estimated by Tang et al. (2025) [7]. A Put stock solution (10^−3^ M) was prepared using sterile distilled water from high-purity Put (≥99% purity; Sigma-Aldrich, Shanghai, China). This stock was then diluted to achieve final concentrations of 0 (control), 10^−7^, 10^−6^, 10^−5^, 10^−4^, and 10^−3^ M, a concentration range established as effective for Put in microalgae and plants [18,27,28]. The experiments were carried out in 1 L Erlenmeyer flasks with a working volume of 600 mL. All cultures, starting at an initial biomass of 0.1 g L^−1^, were maintained under continuous light (250 μmol photons m^−2^ s^−1^) and shaken at 110 rpm. Sampling for the analysis of dry cell weight (DCW), β-carotene, photosynthetic pigments, and photosynthetic activity was performed at two-day intervals. The photosynthetic rates presented in this study were specifically measured on Day 4, as this time point represented a key phase of active growth and stress response across the temperature regimes.

### 2.3. Analytical Methods

#### 2.3.1. Biomass Quantification

DCW was determined by centrifuging 10 mL of culture at 3000 rpm for 2 min, washing the pellet twice with 0.9% (*w*/*v*) NaCl to prevent osmotic lysis, and drying the biomass at 105 °C until a constant weight was achieved [26].

#### 2.3.2. Chlorophyll Quantification

Chlorophyll profiles were quantified via methanol extraction, following the protocol of Tang et al. (2025) [7]. Briefly, 4 mL of microalgal culture was centrifuged at 12,000× *g* for 5 min to harvest the cells. The resulting pellet was resuspended in 4 mL of methanol, vigorously vortexed, and then subjected to dark incubation at 60 °C for 50 min. Following incubation, the sample was centrifuged at 10,000× *g* for 3 min. The absorbance of the supernatant was measured at 653 nm and 666 nm using a UV-5100 spectrophotometer (Shanghai Yuan Analytical Instrument Co., Ltd., Shanghai, China). Pigment concentrations (mg L^−1^) were calculated using Equations (1)–(3), and Chlorophyll content (mg g^−1^ DCW) was calculated using Equation (4).

Chl a = 15.65 A_666_ − 7.34 A_653_ (1)

Chl b = 27.07 A_653_ − 111.21 A_666_ (2)

Chl = Chl a + Chl b(3)

Chl content = Chl/DCW(4)

where A_x_ represents the absorbance at wavelength x, and DCW is the dry cell weight (g L^−1^).

#### 2.3.3. β-Carotene Quantification

β-carotene concentration was quantified following the method of Xi et al. (2022) [26]. In brief, a 1 mL sample of cell suspension was centrifuged (10,000× *g*, 2 min), and the resulting pellet was resuspended in 3 mL of dodecane and vortexed for 20 s (XW-80A, Shanghai Chitang Electronics Co., Ltd., Shanghai, China). To lyse the cells, 9 mL of methanol was added to the mixture, which was then vortexed for another 20 s. After phase separation by centrifugation under the same conditions, the upper dodecane layer containing the extracted β-carotene was collected. The absorbance was measured at 453 nm and 665 nm against a pure dodecane blank. The β-carotene concentration (mg L^−1^) was calculated using Equation (5):

β-carotene = (A_453_ − A_666_/3.91) × 3.657 × 3 × X (5)

where (A_453_ − A_665_/3.91) corresponds to the absorbance corrected for β-carotene; 3.657 is the HPLC-derived calibration factor; 3 represents the volume of dodecane used for extraction (mL); and X denotes the applicable dilution factor.

#### 2.3.4. Photosynthetic Activity

Photosynthetic activity was assessed using a dissolved-oxygen (DO) monitoring system (Fibox 4, PreSens Precision Sensing GmbH, Regensburg, Germany). The system was configured with temperature-controlled chambers, 50 mL clear and opaque vessels, magnetic stirrers, and an LED light source that simulated the culture conditions. Microalgal samples (50 mL) were placed in the measurement vessels and allowed to acclimate for 10 min at the respective assay temperatures of 28 °C, 34 °C, and 40 °C. The change in DO concentration was monitored over time under light for the net photosynthetic rate (P_n_) and in darkness for the respiratory rate (R). The gross photosynthetic rate (P_g_) was derived from these measurements. All rates (μmol O_2_ 10^−7^ cells min^−1^) were calculated using Equations (6)–(8):

P_n_ = (10^−3^ × ∆DO_1_)/(DCW × N × t) (6)

R = (10^−3^ × ∆DO_2_)/(DCW × N × t) (7)

P_g_ = P_n_ + R (8)

where ΔDO_1_ is the DO change (mg L^−1^) during illumination, ΔDO_2_ is the DO change (mg L^−1^) during darkness, t is the duration of measurement (min), N is the cell concentration (10^7^ cells mL^−1^), and DCW is the dry cell weight (g L^−1^).

#### 2.3.5. Chlorophyll Fluorescence

A 4 mL cell sample was dark-adapted for 15 min. Chlorophyll fluorescence parameters were measured using an Aquapen fluorometer (AP110-C, PSI Inc., Drasov, Czech Republic). Key parameters reflecting PSII function included Fv/Fm (maximum photochemical quantum yield), Y(II) (effective quantum yield of PSII photochemistry), NPQ (non-photochemical quenching), φ_EO_ (quantum yield for electron transport), δ_RO_ (efficiency of end acceptor reduction at PS I), and PI_ABS_ (performance index based on absorption).

#### 2.3.6. Intracellular NO Analysis

Intracellular NO level was quantified fluorometrically using the specific fluorescent probe 4-amino-5-methylamino-2′,7′-difluorofluorescein diacetate (DAF-FM DA), following a protocol adapted from Li et al. (2020) [29] with slight modifications. In brief, cell aliquots equivalent to 1 × 10^6^ cells were collected by centrifugation at 10,000× *g* for 2 min and washed twice with ice-cold phosphate-buffered saline (PBS; 0.1 M, pH 7.0). The cell pellet was then incubated with 5 µM DAF-FM DA in PBS (1 mL final volume) at 37 °C in the dark for 1 h, with gentle mixing at 5-min intervals. After incubation, the cells were centrifuged and washed twice with PBS to remove excess extracellular probe. The final pellet was resuspended in 1 mL PBS, and the fluorescence intensity was measured using a SpectraMax M2e microplate reader (Molecular Devices, San Jose, CA, USA) with excitation and emission wavelengths set at 495 nm and 515 nm, respectively. The results were presented as relative fluorescence units per 10^6^ cells.

#### 2.3.7. Antioxidative Responses

Oxidative stress and antioxidative defense parameters were quantified using commercial assay kits (Beyotime Biotechnology Co., Ltd., Shanghai, China) following the manufacturer’s protocols. These parameters included ROS levels (ROS, superoxide anion (O_2_^−^) production rate, and hydrogen peroxide (H_2_O_2_) concentration), antioxidant enzyme activities (superoxide dismutase (SOD), catalase (CAT), and peroxidase (POD)), non-enzymatic antioxidant contents (glutathione (GSH) and ascorbic acid (ASA)), and malondialdehyde (MDA) content as a marker of lipid peroxidation.

#### 2.3.8. Statistical Analysis

All data are presented as the means ± standard deviation (SD) from three independent biological replicates. The effects of temperature, Put concentration, and their interaction on all measured parameters were analyzed using a two-way analysis of variance (ANOVA). When a significant interaction was found, Tukey’s Honest Significant Difference (HSD) post hoc test was applied for multiple comparisons among treatment groups.

## 3. Results

### 3.1. Put Modulates the Biomass–β-Carotene Trade-Off Through Temperature-Dependent Mechanisms

**Figure 1:**
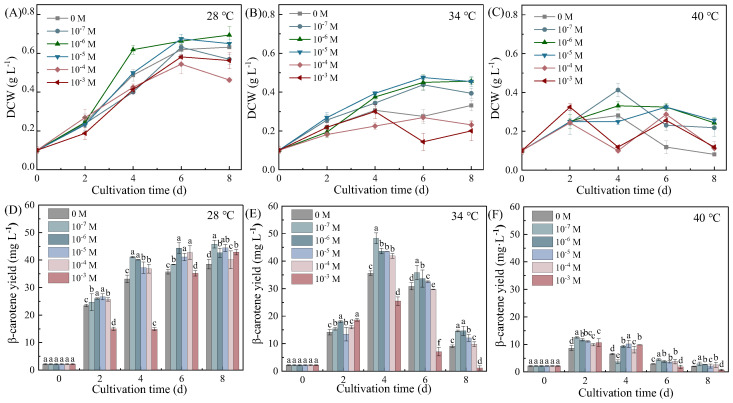
Effects of Put on DCW (**A**–**C**) and β-carotene yield (**D**–**F**) in *Dunaliella salina* under different temperature conditions. (Full statistical results (two-way ANOVA and post-hoc tests) are available in Tables S1–S5. Significant differences among Put concentrations at the same temperature in panels (**D**–**F**) are directly indicated in the figure. Different letters indicate significant differences (*p* < 0.05), while identical letters indicate no significant differences (*p* > 0.05). The same applies to subsequent figures).

Exogenous Put differentially modulated biomass accumulation and β-carotene synthesis in *Dunaliella salina* across thermal regimes, revealing distinct temperature-adaptive strategies to alleviate biomass–β-carotene trade-off (Figure 1). Two-way ANOVA analysis revealed that although both temperature and Put significantly affected DCW and β-carotene yield, with temperature being the stronger factor, their interaction was significant for β-carotene yield but not for DCW (Tables S1 and S4).

At 28 °C, 10^−6^ M Put enhanced biomass accumulation by 9.52% (0.69 g L^−1^) and β-carotene yield by 10.72% (42.65 mg L^−1^) on Day 8. The significant interaction between temperature and Put was clearly manifested at 34 °C, where 10^−7^ M Put induced a synergistic peak in biomass and β-carotene synthesis by Day 4. Biomass increased by 9.68% (0.34 g L^−1^) and β-carotene yield surged by 35.71% (48.34 mg L^−1^) on Day 4, representing the highest β-carotene productivity observed (11.55 mg L^−1^ d^−1^). This divergence in accumulation patterns underscores the distinct temperature strategies: the transient peak at 34 °C reflects rapid, stress-induced biosynthesis, whereas the higher final yield at 28 °C results from sustained accumulation coupled with prolonged growth. In contrast, the absence of significant interaction for DCW was consistent with the more uniform effect of Put on growth across temperatures. Under extreme thermal stress (40 °C), Put-treated cultures maintained higher β-carotene levels despite substantial growth suppression. Specifically, by Day 2, 10^−7^ M Put increased β-carotene yield by 44.99% relative to the control, while biomass dropped by 2.50%, further highlighting the temperature-dependent functional shift of Put toward photoprotection under extreme thermal stress.

### 3.2. Temperature- and Concentration-Dependent Regulation of Photosynthetic Pigments and Carbon Fixation by Put

**Figure 2:**
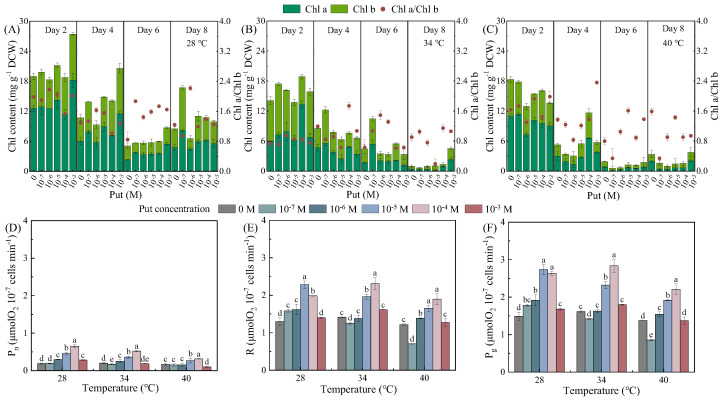
Effects of Put on Chlorophyll content (**A**–**C**), net photosynthetic rate (P_n_, **D**), respiration rate (R, **E**), and gross photosynthetic rate (P_g_, **F**) in *Dunaliella salina* under different temperature conditions. (Full statistical results (two-way ANOVA and post-hoc tests) are available in Tables S6–S16. Significant differences among Put concentrations at the same temperature in panels (**D**–**F**) are directly indicated in the figure).

Exogenous Put regulated photosynthetic pigment dynamics and carbon fixation in *Dunaliella salina* in a temperature- and concentration-dependent manner (Figure 2). Two-way ANOVA analysis revealed significant effects of both temperature and Put on Chl a and Chl b content, with temperature being the dominant factor, along with a significant interaction between the two factors (Tables S6 and S9). In contrast, for the Chl a/Chl b ratio, significant effects were confined to Day 2, where only temperature had a significant influence (Table S12).

At 28 °C, total Chl content in the control group decreased by 55.09% from Day 2 to Day 8, while moderate Put treatments (10^−6^–10^−4^ M) effectively mitigated heat-induced pigment loss. Specifically, on Day 4, Put-treated groups exhibited marked increases in Chl a (92.01%), Chl b (91.95%), total Chl (86.78%), and Chl a/Chl b (26.36%) ratios compared to the control (Figure 2A–C). For photosynthetic carbon fixation parameters, two-way ANOVA analysis indicated that both temperature and Put significantly affected P_n_, R, and P_g_, with Put exhibiting a stronger effect than temperature (Table S15). Significant interactions between the two factors were also observed for all three parameters. Correspondingly, P_n_ and P_g_, and R were significantly improved on Day 4, with P_n_ increasing by 132.14% at 10^−4^ M, P_g_ by 62.79% at 10^−5^ M, and R by 62.86% at 10^−5^ M.

At 34 °C, Put exerted pronounced protective effects on chlorophyll stability. Control cultures experienced a 92.91% decline in total Chl by Day 8, whereas 10^−7^ M Put treatment increased total Chl content by 43.16%. These structural effects translated into functional improvements, with P_g_ and P_n_ at 10^−4^ M elevated by 75.54% and 160.00%, respectively. In contrast, both low (10^−7^ M) and high (10^−3^ M) Put concentrations impaired photosynthetic performance.

At 40 °C, 10^−4^ M Put partially mitigated heat-induced pigment degradation and sustained photosynthetic capacity. While the control group lost 71.20% of total Chl content by Day 4, Put-treated cultures retained significantly higher pigment levels (11.68 vs. 5.27 mg L^−1^, a 121.63% increase). Notably, the observed decline in Chl content alongside the rise in β-carotene (Figure 1F) illustrates a metabolic reallocation from light-harvesting to photoprotection. This strategic shift reduces excitation pressure on photosystems and enhances energy dissipation capacity, thereby directly facilitating β-carotene synthesis under stress. Cultures showed the highest P_g_ (2.21 μmol O_2_·mg^−1^·min^−1^) and P_n_ (0.32 μmol O_2_·mg^−1^·min^−1^) values. The maintenance of carbon fixation despite substantial pigment loss suggests a Put-induced prioritization toward protecting the functional core of the photosynthetic apparatus. Conversely, 10^−3^ M Put suppressed P_g_ by 39.39%, and 10^−7^ M Put impaired pigment biosynthesis, with total Chl content decreasing by 35.67% on Day 4.

### 3.3. Temperature-Specific Modulation of Photochemical Efficiency by Put

**Figure 3:**
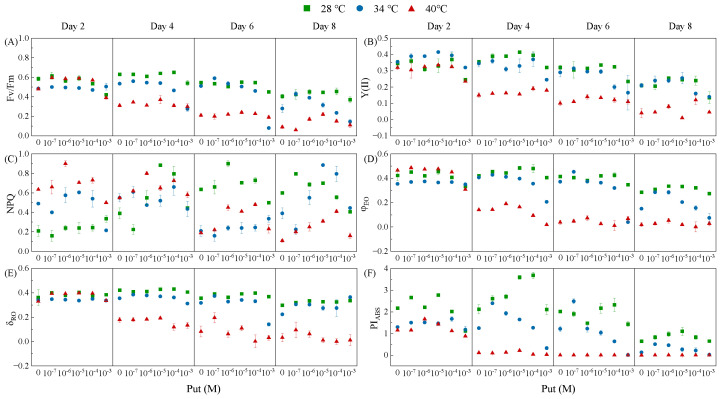
Effects of Put on major chlorophyll fluorescence parameters in *Dunaliella salina* under different temperature conditions. (**A**) Fv/Fm; (**B**) Y(II); (**C**) NPQ; (**D**) φ_EO_; (**E**) δ_RO_; (**F**) PI_ABS_. (Full statistical results (two-way ANOVA and post-hoc tests) are available in Tables S17–S34).

Chlorophyll fluorescence analysis revealed that Put regulates photosynthetic performance in *Dunaliella salina* in a temperature-dependent manner (Figure 3), providing mechanistic insights into the functional protection of the photosynthetic apparatus inferred from the data in Figure 2. Two-way ANOVA analysis demonstrated that both temperature and Put significantly influenced all major chlorophyll fluorescence parameters, with temperature exhibiting a stronger overall effect than Put (Tables S17, S20, S23, S26, S29, and S32). Notably, significant interactions between temperature and Put were observed for Fv/Fm, Y(II), NPQ, φ_EO_, and PI_ABS_ throughout the entire induction period. For δ_RO_, however, a significant interaction emerged only during the later induction phase (Day 6 and Day 8).

At moderate temperatures (28 °C and 34 °C), optimal Put concentrations significantly enhanced photochemical efficiency. Specifically, at 28 °C, 10^−5^ M Put on Day 2 increased PI_ABS_ and φ_EO_ by 27.65% and 7.14%, respectively. At 34 °C, 10^−7^ M Put on Day 8 elevated Fv/Fm by 53.57% relative to the control. These enhancements indicate Put-mediated stabilization of PSII and improved electron transport efficiency. Under extreme thermal stress (40 °C), the functional role of Put shifted toward photoprotection. Notably, 10^−4^ M Put increased NPQ and δ_RO_ by 24.96% and 19.92% compared to the control, respectively, on Day 2, indicating enhanced thermal dissipation of excess excitation energy and sustained Q_A_^−^ reoxidation beyond PSII. This direct evidence of preserved and enhanced δ_RO_ and NPQ under Put treatment at 40 °C provides the functional explanation for the sustained carbon fixation observed despite chlorophyll loss in Figure 2F. Y(II) was also partially maintained under this treatment, suggesting that Put preserved a fraction of PSII photochemical efficiency despite heat-induced damage.

In contrast, supraoptimal concentrations (10^−3^ M) consistently suppressed fluorescence parameters across all temperatures. At 40 °C, 10^−3^ M Put reduced Y(II) and PI_ABS_ by 26.56% and 24.14%, respectively, on Day 2, reflecting the cytotoxic impairment of PSII functionality.

### 3.4. Temperature-Stratified Modulation of Antioxidant Systems by Put

**Figure 4:**
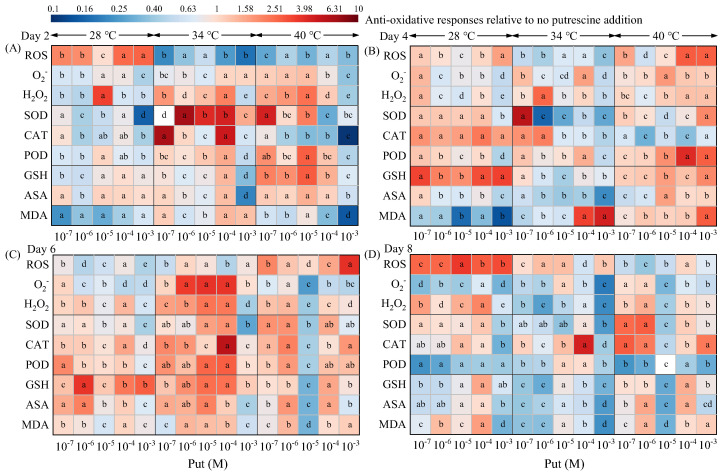
Effects of Put on antioxidative responses in *Dunaliella salina* under different temperature conditions. (Full statistical results (two-way ANOVA and post-hoc tests) are available in Tables S35–S52. Significant differences among Put concentrations at the same temperature of panels (**A**–**D**) are directly indicated in the figure).

Exogenous Put orchestrated a hierarchical restructuring of antioxidant defenses in *Dunaliella salina* under different temperature regimes (Figure 4). The oxidative stress and antioxidant response were significantly influenced by both temperature and Put. Statistical analysis confirmed that temperature was the dominant factor, and significant interactions between the two factors were observed throughout the coordinated antioxidant defense (Tables S35, S37, S39, S41, S43, S45, S47, S49, and S51).

At 28 °C, Put triggered a biphasic antioxidant response. In the early phase (Day 4), 10^−6^ M Put increased SOD, CAT, POD, and GSH levels by 41.09%, 78.29%, 22.73%, and 138.83% while reducing ROS, O_2_^−^, H_2_O_2_, and MDA levels by 29.58%, 22.09%, 11.40%, and 51.69%. However, MDA levels increased by 6.77% relative to the controls by Day 6, likely attributable to polyamine oxidase (PAO) activation that triggered a concomitant 1.45-fold H_2_O_2_ surge [30,31]. This ROS accumulation on Day 6 was accompanied by compensatory increases in non-enzymatic antioxidants (GSH and ASA contents increased 4.15- and 2.38-fold, respectively).

At 34 °C, 10^−7^ M Put induced a coordinated antioxidant response by Day 4. Coordinated upregulation of SOD (6.06-fold), CAT (2.15-fold), and POD (1.46-fold) coupled with GSH elevation (1.17-fold) reduced MDA levels by 43.90%. Importantly, this antioxidant priming permitted controlled ROS bursts, with O_2_^−^ and H_2_O_2_ increasing by 56.03% and 11.22%, respectively. These temporal peaks closely correlated with maximal β-carotene accumulation (Figure 1E).

At 40 °C, antioxidant defenses were rapidly overwhelmed despite early antioxidant defense system activation. On Day 2, 10^−7^ M Put increased SOD (5.02-fold), POD (2.19-fold), GSH (2.38-fold), and ASA (1.37-fold) levels and reduced ROS (by 76.72%) and MDA (by 25.76) levels. However, by Day 4, MDA levels in the 10^−7^ M Put treatment had risen by 8.97% relative to the control, indicating that Put had shifted from acting as an antioxidant to functioning as a pro-oxidant.

### 3.5. NO as a Central Mediator of Put-Induced Thermal Adaptation

**Figure 5:**
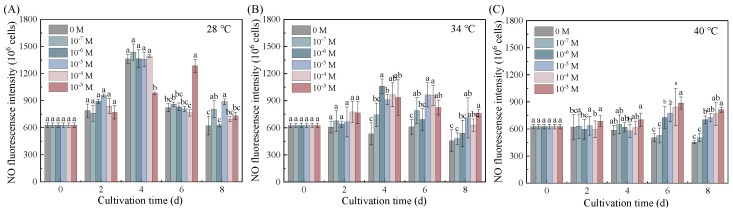
Effects of Put on NO levels in *Dunaliella salina* under different temperature conditions. (Full statistical results (two-way ANOVA and post-hoc tests) are available in Tables S53 and S54. Significant differences among Put concentrations at the same temperature of panels (**A**–**C**) are directly indicated in the figure).

Put enhanced thermotolerance in *Dunaliella salina* through pathways involving NO that integrate growth, photoprotection, and redox homeostasis (Figure 5). Two-way ANOVA analysis confirmed that NO levels were significantly influenced by both temperature and Put, with temperature as the predominant factor and a significant interaction between them (Table S53). The accumulation kinetics of NO exhibited temperature dependence, with the earliest peak observed at 40 °C (Day 2) and the latest at 28 °C (Day 6). This statistical interaction was particularly evident at 34 °C, where 10^−7^ M Put elevated NO levels by 1.99-fold on Day 4. This peak coincided temporally with maximal β-carotene accumulation (48.34 mg L^−1^). Concurrent responses included the preservation of photosynthetic efficiency (Fv/Fm maintained at 0.56), a 43.90% reduction in MDA, and coordinated upregulation of antioxidant enzymes.

This NO surge likely functioned as both a redox buffer and a signaling modulator [32]. The temporal association of NO surge with β-carotene peak implies coordinated stress adaptation, though direct mechanistic links require further validation. Notably, NO accumulation preceded antioxidant activation by 24–48 h, a temporal sequence that is consistent with a potential upstream signaling role for NO in the stress response cascade.

Under extreme thermal stress (40 °C), the functional consequence of the temperature–Put interaction shifted, with 10^−7^ M Put inducing only a transient NO elevation (an 8.05% increase on Day 2), insufficient to sustain protective responses. Thermal disruption of NO synthase systems likely caused a collapse of the Put–NO signaling network, culminating in progressive NO decline through Day 8.

## 4. Discussion

The results of this study demonstrate that exogenous Put alleviates the biomass–β-carotene trade-off in *Dunaliella salina* through temperature-dependent mechanisms. At 28 °C (optimal for growth), Put enhanced both biomass and β-carotene yield, suggesting an optimized carbon partitioning between growth and secondary metabolism under non-stress conditions, likely by accelerating electron transport and, relative to the control, mitigating the heat-induced degradation of photosynthetic chlorophyll complexes. This is consistent with the known role of Put in promoting cell division in microalgae such as *Haematococcus pluvialis* [15]. At 34 °C (optimal for β-carotene synthesis), a low concentration of Put induced a synergistic increase in both biomass and β-carotene on Day 4, implying that Put activates stress-responsive pathways that balance growth with photoprotective carotenogenesis. This aligns with findings in heat-stressed plants where polyamines co-regulate biomass and secondary metabolite biosynthesis [33]. Under extreme thermal stress (40 °C), Put treatment maintained significantly higher β-carotene levels despite biomass suppression, indicating a regulatory shift toward photoprotective metabolism.

The impact of Put on the photosynthetic apparatus was reflected in its temperature- and concentration-dependent regulation of photosynthetic pigment stability and carbon fixation capacity. At 28 °C and 34 °C, Put effectively mitigated chlorophyll degradation. This may be partially attributed to Put catabolism providing nitrogen substrates for chlorophyll biosynthesis, as both pathways share glutamate as a common precursor [34]. The corresponding enhancement in photosynthetic rates and the positive effects on the stability of PSII core complexes under moderate stress correspond with reports of Put maintaining photosynthetic membrane integrity in higher plants [24]. At 40 °C, the partial maintenance of carbon fixation capacity despite pigment degradation, coupled with the fluorescence-based evidence of enhanced NPQ and δ_RO_ (Figure 3), suggests that Put’s protective focus shifted toward components of the electron transport chain rather than light-harvesting complexes, consistent with findings by Jahan et al. (2022) [16]. This hierarchical protection strategy—safeguarding core photochemical function over light-harvesting structures—explains the apparent disconnect between chlorophyll content and photosynthetic performance and is a key temperature-dependent aspect of Put’s efficacy. However, the inhibitory effects of supraoptimal Put concentrations underscore the critical importance of dose optimization.

Put orchestrated a hierarchical strategy in modulating the antioxidant system. The biphasic antioxidant response at 28 °C, characterized by initial antioxidant enhancement followed by a later increase in H_2_O_2_ and MDA, was likely due to PAO activation, revealing the dual role of Put in microalgae as both an antioxidant and a precursor for ROS signaling via the PAO pathway [30,31]. At 34 °C, Put coordinated the upregulation of antioxidant enzymes and glutathione accumulation, effectively reducing MDA content while permitting a controlled ROS burst. The temporal coincidence of this ROS peak with maximum β-carotene accumulation suggests that Put might facilitate a moderate oxidative environment that activates ROS-mediated signaling for carotenogenesis. Based on these spatial and temporal dynamics, we propose a mechanistic pathway for Put action: exogenous Put is likely metabolized by PAO, generating H_2_O_2_ as a key signaling molecule. This PAO-derived H_2_O_2_ then functions as a controlled oxidative signal that, in coordination with NO, activates downstream stress-responsive pathways. At the optimal stress level (34 °C), this signaling network precisely upregulates carotenogenic genes and enhances antioxidant capacity, thereby achieving the synergistic improvement in biomass and β-carotene yield. In contrast, under extreme thermal stress (40 °C), the antioxidant defenses were rapidly overwhelmed despite early activation, and the Put-induced signaling cascade collapsed into uncontrolled oxidation, indicating a thermal threshold beyond its protective capacity.

The dynamics of NO support its potential role as a key component in the Put signaling pathway. At 34 °C, the Put-induced peak in NO accumulation coincided temporally with the maxima in β-carotene accumulation, preservation of photosynthetic efficiency, and coordinated activation of antioxidant defenses. The precedence of NO accumulation over the full activation of antioxidant enzymes is consistent with a potential upstream signaling role, consistent with observations in *Chlorella* sp. [35]. This temporal pattern suggests that NO may act as an intracellular messenger integrating with ROS and Ca^2+^ cascades to initiate thermotolerance mechanisms [36]. At 40 °C, the weak and transient nature of the NO signal, and the subsequent collapse of the Put–NO signaling network, define the upper limit of Put’s protective capacity. The collective NO dynamics—ranging from a transient pulse at 28 °C to a sustained peak at 34 °C and a collapsed signal at 40 °C—collectively demonstrate that the efficacy and fate of Put-induced NO signaling are intrinsically dependent on the cellular energetic and redox state dictated by the severity of thermal stress.

It is important to note that the findings of this study are based on a specific microalgal strain (*Dunaliella salina* CCAP 19/18), and thus the observed responses may be strain-specific. Compared to other plant growth regulators applied in *Dunaliella salina*, such as melatonin [11] and 24-epibrassinolide [8], Put offers the advantages of low cost, high membrane permeability, and multifunctional regulatory effects. Furthermore, the potential environmental concerns associated with its application are minimal. The optimal concentrations identified (10^−7^ to 10^−6^ M) are exceptionally low and non-toxic, functioning as signaling triggers rather than pollutants. Coupled with the inherent biodegradability of putrescine in the environment, these factors underscore its promise as a sustainable and eco-compatible strategy for large-scale microalgal cultivation. In conclusion, Put, through its temperature-dependent, coordinated modulation of photosystem plasticity, antioxidant defenses, and NO signaling, effectively alleviates the biomass–β-carotene production conflict in *Dunaliella salina*. The temperature–concentration framework established in this study provides a practical strategy for regulating microalgal cultivation under fluctuating environmental conditions. The temperature-dependent efficacy of Put provides a scalable strategy to mitigate seasonal fluctuations in β-carotene yield during outdoor cultivation, by adjusting application concentrations to match prevailing thermal conditions.

## 5. Conclusions

This study establishes Put as a temperature-responsive regulator that alleviates the biomass–β-carotene trade-off in *Dunaliella salina*. The efficacy and mechanism of Put action were highly temperature-dependent, progressing from enhanced photosynthetic capacity at 28 °C, through coordinated NO-associated redox signaling that boosted both biomass and β-carotene at 34 °C, to prioritized photoprotection sustaining β-carotene under extreme 40 °C stress. These effects were underpinned by coordinated modulation of photosystem plasticity, antioxidant defenses, and putative NO-dependent processes. These findings provide a practical temperature–concentration framework for applying Put to enhance β-carotene production in microalgae under fluctuating thermal environments.

## Data Availability

Data will be made available on request from the corresponding author.

## Supplementary Materials

The following supporting information can be downloaded at: https://www.mdpi.com/article/10.3390/life15121807/s1.

## Abbreviations

The following abbreviations are used in this manuscript:

Put	Putrescine
NO	Nitric oxide
ROS	Reactive oxygen species
NPQ	Non-photochemical quenching
PGRs	Plant growth regulators
PAs	Polyamines
DCW	Dry cell weight
DO	Dissolved oxygen
P_n_	Net photosynthetic rate
R	Respiratory rate
P_g_	Gross photosynthetic rate
O_2_^−^	Superoxide anion
H_2_O_2_	Hydrogen peroxide
SOD	Superoxide dismutase
CAT	Catalase
POD	Peroxidase
GSH	Glutathione
ASA	Ascorbic acid
MDA	Malondialdehyde
PAO	Polyamine oxidase
