# Supplementary material for: Putrescine Mitigates the Biomass–β-Carotene Conflict in Dunaliella salina Under Thermal Stress
Source: Life (Basel). 2025 Nov 25;15(12):1807. doi: 10.3390/life15121807 (PMC12734997; doi:10.3390/life15121807)
Supplement: Supplementary file 1 [file life-15-01807-s001.zip › life-3975307-supplementary.docx]

**Table S1.** Two-way ANOVA results for the effects of temperature and putrescine concentration on DCW.

| Factors | Day 2 | | Day 4 | | Day 6 | | Day 8 | |
| --- | --- | --- | --- | --- | --- | --- | --- | --- |
|  | F | *p* | F | *p* | F | *p* | F | *p* |
| Temperature | 19.701 | <0.001 | 1.362 | 0.092 | 25.109 | <0.001 | 139.49 | <0.001 |
| Putrescine | 0.891 | 0.092 | 0.130 | 0.092 | 4.979 | <0.01 | 8.890 | <0.001 |
| Temperature* Putrescine | 1.268 | 0.092 | 0.835 | 0.092 | 1.522 | 0.092 | 1.939 | 0.092 |

**Table S2.** Post-hoc comparisons of the temperature effect on DCW within each putrescine concentration.

| Dunnett's multiple comparisons test | | Summary | | | | Adjusted *p* value | | | |
| --- | --- | --- | --- | --- | --- | --- | --- | --- | --- |
|  |  | Day 2 | Day 4 | Day 6 | Day 8 | Day 2 | Day 4 | Day 6 | Day 8 |
| 0 M | 28 ℃ vs. 34 ℃ | ns | ns | * | ** | 0.9179 | 0.0625 | 0.0476 | 0.0077 |
|  | 28 ℃ vs. 40 ℃ | ns | * | * | ** | 0.9945 | 0.0448 | 0.0172 | 0.0013 |
|  | 34 ℃ vs. 40 ℃ | ns | ns | ns | * | 0.8766 | 0.8639 | 0.2739 | 0.0129 |
| 10^-7^ M | 28 ℃ vs. 34 ℃ | ns | ns | * | * | 0.9073 | 0.1458 | 0.0403 | 0.0146 |
|  | 28 ℃ vs. 40 ℃ | ns | ns | ** | ** | 0.9889 | 0.8330 | 0.0053 | 0.0017 |
|  | 34 ℃ vs. 40 ℃ | ns | ns | * | * | 0.9569 | 0.0923 | 0.0341 | 0.0108 |
| 10^-6^ M | 28 ℃ vs. 34 ℃ | ns | ns | ns | * | 0.8525 | 0.3456 | 0.2947 | 0.0251 |
|  | 28 ℃ vs. 40 ℃ | ns | ns | ns | ** | 0.9974 | 0.2636 | 0.1175 | 0.004 |
|  | 34 ℃ vs. 40 ℃ | ns | ns | ns | * | 0.8194 | 0.9520 | 0.5809 | 0.0332 |
| 10^-5^ M | 28 ℃ vs. 34 ℃ | ns | ns | ns | ns | 0.2673 | 0.6646 | 0.3312 | 0.0525 |
|  | 28 ℃ vs. 40 ℃ | ns | ns | ns | ** | 0.6350 | 0.2206 | 0.1111 | 0.0078 |
|  | 34 ℃ vs. 40 ℃ | ns | ns | ns | ns | 0.6350 | 0.5039 | 0.4880 | 0.0534 |
| 10^-4^ M | 28 ℃ vs. 34 ℃ | ns | ** | * | ns | 0.0902 | 0.0017 | 0.0377 | 0.1445 |
|  | 28 ℃ vs. 40 ℃ | ns | *** | * | ns | 0.6549 | 0.0004 | 0.0454 | 0.0976 |
|  | 34 ℃ vs. 40 ℃ | ns | ** | ns | ns | 0.1897 | 0.0066 | 0.9469 | 0.8731 |
| 10^-3^ M | 28 ℃ vs. 34 ℃ | ns | * | ** | ns | 0.8842 | 0.0345 | 0.0045 | 0.055 |
|  | 28 ℃ vs. 40 ℃ | ns | ** | * | * | 0.2321 | 0.0023 | 0.0106 | 0.0399 |
|  | 34 ℃ vs. 40 ℃ | ns | ** | ns | ns | 0.3575 | 0.0091 | 0.1619 | 0.8642 |

*indicates *p*<0.05; **indicates *p*<0.01; ***indicates *p*<0.001; ****indicates *p*<0.0001; ns indicates not significant.

**Table S3.** Post-hoc comparisons of the putrescine concentration effect on DCW within each temperature.

| Dunnett's multiple comparisons test | | Summary | | | | Adjusted *p* value | | | |
| --- | --- | --- | --- | --- | --- | --- | --- | --- | --- |
|  |  | Day 2 | Day 4 | Day 6 | Day 8 | Day 2 | Day 4 | Day 6 | Day 8 |
| 28 ℃ | Control vs. 10^-7^ M | ns | * | ns | ns | >0.9999 | 0.0245 | 0.9981 | 0.3599 |
|  | Control vs. 10^-6^ M | ns | ** | ns | ns | >0.9999 | 0.0032 | 0.7381 | 0.3599 |
|  | Control vs. 10^-5^ M | ns | ns | ns | ns | 0.9986 | 0.9856 | 0.5386 | 0.9811 |
|  | Control vs. 10^-4^ M | ns | ns | ns | ** | 0.9686 | 0.1000 | 0.2965 | 0.0080 |
|  | Control vs. 10^-3^ M | ns | * | ns | ns | 0.5919 | 0.0484 | 0.8310 | 0.2850 |
|  | 10^-7^ M vs. 10^-6^ M | ns | *** | ns | * | >0.9999 | 0.0002 | 0.9072 | 0.0334 |
|  | 10^-7^ M vs. 10^-5^ M | ns | * | ns | ns | >0.9999 | 0.0136 | 0.7381 | 0.1756 |
|  | 10^-7^ M vs. 10^-4^ M | ns | ns | ns | ns | 0.9257 | 0.7594 | 0.1918 | 0.0666 |
|  | 10^-7^ M vs. 10^-3^ M | ns | ns | ns | ns | 0.6866 | 0.9795 | 0.6377 | 0.9999 |
|  | 10^-6^ M vs. 10^-5^ M | ns | ** | ns | ns | 0.9986 | 0.0052 | 0.9981 | 0.6626 |
|  | 10^-6^ M vs. 10^-4^ M | ns | *** | ns | ** | 0.9686 | 0.0004 | 0.0642 | 0.0015 |
|  | 10^-6^ M vs. 10^-3^ M | ns | *** | ns | * | 0.5919 | 0.0003 | 0.2389 | 0.0268 |
|  | 10^-5^ M vs. 10^-4^ M | ns | ns | * | ** | 0.8610 | 0.0512 | 0.0423 | 0.0046 |
|  | 10^-5^ M vs. 10^-3^M | ns | * | ns | ns | 0.7789 | 0.0258 | 0.1538 | 0.1374 |
|  | 10^-4^ M vs. 10^-3^ M | ns | ns | ns | ns | 0.2832 | 0.9795 | 0.8310 | 0.0845 |
| 34 ℃ | Control vs. 10^-7^ M | ns | ns | * | ns | 0.6364 | 0.4493 | 0.0163 | 0.4079 |
|  | Control vs. 10^-6^ M | ns | ns | * | * | 0.8836 | 0.0749 | 0.0114 | 0.0429 |
|  | Control vs. 10^-5^ M | ns | * | ** | * | 0.3901 | 0.0268 | 0.0058 | 0.0458 |
|  | Control vs. 10^-4^ M | ns | * | ns | ns | 0.6364 | 0.0374 | >0.9999 | 0.1048 |
|  | Control vs. 10^-3^ M | ns | ns | * | * | >0.9999 | 0.9992 | 0.0432 | 0.0347 |
|  | 10^-7^ M vs. 10^-6^ M | ns | ns | ns | ns | 0.2211 | 0.6066 | 0.9981 | 0.4079 |
|  | 10^-7^ M vs. 10^-5^ M | ns | ns | ns | ns | 0.9927 | 0.2240 | 0.8338 | 0.4340 |
|  | 10^-7^ M vs. 10^-4^ M | ns | ** | * | * | 0.1226 | 0.0061 | 0.0136 | 0.0128 |
|  | 10^-7^ M vs. 10^-3^ M | ns | ns | *** | ** | 0.6364 | 0.3205 | 0.0007 | 0.0053 |
|  | 10^-6^ M vs. 10^-5^ M | ns | ns | ns | ns | 0.1226 | 0.9081 | 0.9605 | >0.9999 |
|  | 10^-6^ M vs. 10^-4^ M | ns | ** | ** | ** | 0.9927 | 0.0018 | 0.0096 | 0.0024 |
|  | 10^-6^ M vs. 10^-3^ M | ns | ns | *** | ** | 0.8836 | 0.0526 | 0.0006 | 0.0012 |
|  | 10^-5^ M vs. 10^-4^ M | ns | *** | ** | ** | 0.0685 | 0.0009 | 0.0050 | 0.0025 |
|  | 10^-5^ M vs. 10^-3^M | ns | * | *** | ** | 0.3901 | 0.0195 | 0.0004 | 0.0012 |
|  | 10^-4^ M vs. 10^-3^ M | ns | ns | ns | ns | 0.6364 | 0.0526 | 0.0532 | 0.8915 |
| 40 ℃ | Control vs. 10^-7^ M | ns | ** | * | * | >0.9999 | 0.0031 | 0.0154 | 0.0144 |
|  | Control vs. 10^-6^ M | ns | ns | *** | ** | >0.9999 | 0.2048 | 0.0006 | 0.0063 |
|  | Control vs. 10^-5^ M | ns | ns | *** | ** | >0.9999 | 0.5811 | 0.0006 | 0.0043 |
|  | Control vs. 10^-4^ M | ns | *** | ** | ns | >0.9999 | 0.0005 | 0.0019 | 0.8281 |
|  | Control vs. 10^-3^ M | ns | *** | ** | ns | 0.3005 | 0.0010 | 0.0057 | 0.7142 |
|  | 10^-7^ M vs. 10^-6^ M | ns | * | * | ns | >0.9999 | 0.0327 | 0.0359 | 0.9181 |
|  | 10^-7^ M vs. 10^-5^ M | ns | *** | * | ns | >0.9999 | 0.0010 | 0.0359 | 0.7142 |
|  | 10^-7^ M vs. 10^-4^ M | ns | **** | ns | * | >0.9999 | <0.0001 | 0.2346 | 0.0467 |
|  | 10^-7^ M vs. 10^-3^ M | ns | **** | ns | ns | 0.2425 | <0.0001 | 0.8467 | 0.0602 |
|  | 10^-6^ M vs. 10^-5^ M | ns | * | ns | ns | >0.9999 | 0.0327 | >0.9999 | 0.9954 |
|  | 10^-6^ M vs. 10^-4^ M | ns | *** | ns | * | >0.9999 | 0.0001 | 0.5657 | 0.0180 |
|  | 10^-6^ M vs. 10^-3^ M | ns | *** | ns | * | 0.3005 | 0.0002 | 0.1235 | 0.0226 |
|  | 10^-5^ M vs. 10^-4^ M | ns | ** | ns | * | >0.9999 | 0.0015 | 0.5657 | 0.0116 |
|  | 10^-5^ M vs. 10^-3^M | ns | ** | ns | * | 0.3005 | 0.0031 | 0.1235 | 0.0144 |
|  | 10^-4^ M vs. 10^-3^ M | ns | ns | ns | ns | 0.2425 | 0.8979 | 0.7115 | 0.9998 |

*indicates *p*<0.05; **indicates *p*<0.01; ***indicates *p*<0.001; ****indicates *p*<0.0001; ns indicates not significant.

**Table S4.** Two-way ANOVA results for the effects of temperature and putrescine concentration on β-carotene yield.

| Factors | Day 2 | | Day 4 | | Day 6 | | Day 8 | |
| --- | --- | --- | --- | --- | --- | --- | --- | --- |
|  | F | *p* | F | *p* | F | *p* | F | *p* |
| Temperature | 139.490 | <0.001 | 139.623 | <0.001 | 139.623 | <0.001 | 611.990 | <0.001 |
| Putrescine | 8.890 | <0.001 | 3.570 | <0.05 | 3.570 | <0.05 | 19.241 | <0.001 |
| Temperature* Putrescine | 1.939 | 0.092 | 5.192 | <0.01 | 5.192 | <0.01 | 9.342 | <0.001 |

**Table S5.** Post-hoc comparisons of the temperature effect on β-carotene yield within each putrescine concentration.

| Dunnett's multiple comparisons test | | Summary | | | | Adjusted *p* value | | | |
| --- | --- | --- | --- | --- | --- | --- | --- | --- | --- |
|  |  | Day 2 | Day 4 | Day 6 | Day 8 | Day 2 | Day 4 | Day 6 | Day 8 |
| 0 M | 28 ℃ vs. 34 ℃ | * | ns | * | *** | 0.0277 | 0.1478 | 0.0232 | 0.0001 |
|  | 28 ℃ vs. 40 ℃ | ** | *** | **** | **** | 0.0075 | 0.0001 | <0.0001 | <0.0001 |
|  | 34 ℃ vs. 40 ℃ | ns | **** | **** | * | 0.1072 | <0.0001 | <0.0001 | 0.0124 |
| 10^-7^ M | 28 ℃ vs. 34 ℃ | * | * | ns | **** | 0.0270 | 0.0186 | 0.3493 | <0.0001 |
|  | 28 ℃ vs. 40 ℃ | * | **** | *** | **** | 0.0106 | <0.0001 | 0.0004 | <0.0001 |
|  | 34 ℃ vs. 40 ℃ | ns | **** | *** | ** | 0.2372 | <0.0001 | 0.0006 | 0.0021 |
| 10^-6^ M | 28 ℃ vs. 34 ℃ | *** | * | * | * | 0.0001 | 0.0204 | 0.0309 | 0.0119 |
|  | 28 ℃ vs. 40 ℃ | **** | **** | *** | ** | <0.0001 | <0.0001 | 0.0007 | 0.0043 |
|  | 34 ℃ vs. 40 ℃ | *** | **** | ** | ns | 0.0006 | <0.0001 | 0.0017 | 0.1115 |
| 10^-5^ M | 28 ℃ vs. 34 ℃ | * | * | ** | *** | 0.0323 | 0.0402 | 0.0039 | 0.0001 |
|  | 28 ℃ vs. 40 ℃ | * | *** | **** | **** | 0.0207 | 0.0007 | <0.0001 | <0.0001 |
|  | 34 ℃ vs. 40 ℃ | ns | *** | **** | ** | 0.6925 | 0.0003 | <0.0001 | 0.0062 |
| 10^-4^ M | 28 ℃ vs. 34 ℃ | ns | ns | * | ** | 0.0716 | 0.2014 | 0.0161 | 0.0048 |
|  | 28 ℃ vs. 40 ℃ | * | ** | *** | ** | 0.0195 | 0.0021 | 0.0007 | 0.0026 |
|  | 34 ℃ vs. 40 ℃ | ns | ** | ** | ns | 0.1997 | 0.0013 | 0.0022 | 0.1943 |
| 10^-3^ M | 28 ℃ vs. 34 ℃ | ns | ns | * | *** | 0.0550 | 0.2271 | 0.0260 | 0.0004 |
|  | 28 ℃ vs. 40 ℃ | * | ns | * | *** | 0.0339 | 0.6150 | 0.0162 | 0.0003 |
|  | 34 ℃ vs. 40 ℃ | ** | ns | ns | ns | 0.0062 | 0.0992 | 0.6262 | 0.9674 |

*indicates *p*<0.05; **indicates *p*<0.01; ***indicates *p*<0.001; ****indicates *p*<0.0001; ns indicates not significant.

**Table S6.** Two-way ANOVA results for the effects of temperature and putrescine concentration on Chl a.

| Factors | Day 2 | | Day 4 | | Day 6 | | Day 8 | |
| --- | --- | --- | --- | --- | --- | --- | --- | --- |
|  | F | *p* | F | *p* | F | *p* | F | *p* |
| Temperature | 54.186 | <0.001 | 230.402 | <0.001 | 133.672 | <0.001 | 518.943 | <0.001 |
| Putrescine | 2.190 | 0.092 | 19.271 | <0.001 | 7.345 | <0.001 | 9.421 | <0.001 |
| Temperature* Putrescine | 7.060 | <0.001 | 19.796 | <0.001 | 11.990 | <0.001 | 11.022 | <0.001 |

**Table S7.** Post-hoc comparisons of the temperature effect on Chl a within each putrescine concentration.

| Dunnett's multiple comparisons test | | Summary | | | | Adjusted *p* value | | | |
| --- | --- | --- | --- | --- | --- | --- | --- | --- | --- |
|  |  | Day 2 | Day 4 | Day 6 | Day 8 | Day 2 | Day 4 | Day 6 | Day 8 |
| 0 M | 28 ℃ vs. 34 ℃ | ** | ns | * | ** | 0.0013 | 0.2927 | 0.0194 | 0.0036 |
|  | 28 ℃ vs. 40 ℃ | ns | ns | ** | * | 0.0739 | 0.0510 | 0.0017 | 0.0126 |
|  | 34 ℃ vs. 40 ℃ | ** | ns | ** | ns | 0.0028 | 0.2017 | 0.0090 | 0.0642 |
| 10^-7^ M | 28 ℃ vs. 34 ℃ | * | * | ns | **** | 0.0173 | 0.0361 | 0.0986 | <0.0001 |
|  | 28 ℃ vs. 40 ℃ | ns | ** | * | **** | 0.3389 | 0.0024 | 0.0134 | <0.0001 |
|  | 34 ℃ vs. 40 ℃ | * | ** | ** | ns | 0.0415 | 0.0095 | 0.0044 | 0.9971 |
| 10^-6^ M | 28 ℃ vs. 34 ℃ | * | ns | ns | ** | 0.0485 | 0.1324 | 0.1220 | 0.0020 |
|  | 28 ℃ vs. 40 ℃ | * | * | * | ** | 0.0357 | 0.0183 | 0.0119 | 0.0020 |
|  | 34 ℃ vs. 40 ℃ | ns | ns | ns | ns | 0.8676 | 0.0919 | 0.0502 | 0.9988 |
| 10^-5^ M | 28 ℃ vs. 34 ℃ | * | ** | * | ** | 0.0110 | 0.0065 | 0.0166 | 0.0012 |
|  | 28 ℃ vs. 40 ℃ | ns | ** | ** | ** | 0.0684 | 0.0073 | 0.0031 | 0.0016 |
|  | 34 ℃ vs. 40 ℃ | ns | ns | * | ns | 0.0748 | 0.9339 | 0.0320 | 0.4803 |
| 10^-4^ M | 28 ℃ vs. 34 ℃ | ns | *** | ** | ** | 0.1835 | 0.0008 | 0.0014 | 0.0012 |
|  | 28 ℃ vs. 40 ℃ | ns | ns | **** | ** | 0.2295 | 0.0526 | <0.0001 | 0.0011 |
|  | 34 ℃ vs. 40 ℃ | * | ** | ** | ns | 0.0404 | 0.0016 | 0.0012 | 0.7941 |
| 10^-3^ M | 28 ℃ vs. 34 ℃ | ** | *** | ** | * | 0.0033 | 0.0009 | 0.0011 | 0.0183 |
|  | 28 ℃ vs. 40 ℃ | ** | *** | *** | * | 0.0063 | 0.0010 | 0.0008 | 0.0147 |
|  | 34 ℃ vs. 40 ℃ | ns | ns | ns | ns | 0.2116 | 0.7481 | 0.4111 | 0.8819 |

*indicates *p*<0.05; **indicates *p*<0.01; ***indicates *p*<0.001; ****indicates *p*<0.0001; ns indicates not significant.

**Table S8.** Post-hoc comparisons of the putrescine concentration effect on Chl a within each temperature.

| Dunnett's multiple comparisons test | | Summary | | | | Adjusted *p* value | | | |
| --- | --- | --- | --- | --- | --- | --- | --- | --- | --- |
|  |  | Day 2 | Day 4 | Day 6 | Day 8 | Day 2 | Day 4 | Day 6 | Day 8 |
| 28 ℃ | Control vs. 10^-7^ M | ns | ns | * | ** | 0.9998 | 0.1715 | 0.0194 | 0.0024 |
|  | Control vs. 10^-6^ M | ns | ns | ns | ns | >0.9999 | 0.9983 | 0.0769 | 0.9878 |
|  | Control vs. 10^-5^ M | ns | * | * | ns | 0.7277 | 0.0307 | 0.0476 | 0.2276 |
|  | Control vs. 10^-4^ M | ns | ns | * | ns | 0.8505 | 0.5821 | 0.0309 | 0.1290 |
|  | Control vs. 10^-3^ M | * | ** | *** | ns | 0.0200 | 0.0012 | 0.0003 | 0.5507 |
|  | 10^-7^ M vs. 10^-6^ M | ns | ns | ns | ** | 0.9994 | 0.1117 | 0.7606 | 0.0016 |
|  | 10^-7^ M vs. 10^-5^ M | ns | ns | ns | * | 0.8479 | 0.6304 | 0.9363 | 0.0218 |
|  | 10^-7^ M vs. 10^-4^ M | ns | ns | ns | * | 0.7308 | 0.8350 | 0.9957 | 0.0361 |
|  | 10^-7^ M vs. 10^-3^ M | * | * | ** | ** | 0.0258 | 0.0107 | 0.0066 | 0.0098 |
|  | 10^-6^ M vs. 10^-5^ M | ns | * | ns | ns | 0.7017 | 0.0210 | 0.9966 | 0.1173 |
|  | 10^-6^ M vs. 10^-4^ M | ns | ns | ns | ns | 0.8712 | 0.4053 | 0.9422 | 0.0671 |
|  | 10^-6^ M vs. 10^-3^ M | * | *** | ** | ns | 0.0191 | 0.0009 | 0.0024 | 0.3044 |
|  | 10^-5^ M vs. 10^-4^ M | ns | ns | ns | ns | 0.2456 | 0.1907 | 0.9974 | 0.9939 |
|  | 10^-5^ M vs. 10^-3^M | ns | ns | ** | ns | 0.0855 | 0.0505 | 0.0033 | 0.9443 |
|  | 10^-4^ M vs. 10^-3^ M | ** | ** | ** | ns | 0.0072 | 0.0040 | 0.0045 | 0.7461 |
| 34 ℃ | Control vs. 10^-7^ M | ns | ns | ** | ns | 0.7721 | 0.6453 | 0.0016 | 0.9999 |
|  | Control vs. 10^-6^ M | ns | ns | ns | ns | 0.4464 | 0.6418 | 0.9322 | >0.9999 |
|  | Control vs. 10^-5^ M | ns | ns | ns | ns | 0.9998 | 0.0666 | 0.9961 | 0.9410 |
|  | Control vs. 10^-4^ M | ** | ns | ns | ns | 0.0024 | 0.9995 | 0.9201 | 0.6098 |
|  | Control vs. 10^-3^ M | ns | ns | ns | ** | 0.9566 | 0.3843 | 0.8690 | 0.0044 |
|  | 10^-7^ M vs. 10^-6^ M | ns | ns | ** | ns | 0.9804 | 0.1264 | 0.0030 | >0.9999 |
|  | 10^-7^ M vs. 10^-5^ M | ns | * | ** | ns | 0.8822 | 0.0139 | 0.0022 | 0.9830 |
|  | 10^-7^ M vs. 10^-4^ M | ** | ns | ** | ns | 0.0066 | 0.7912 | 0.0031 | 0.4962 |
|  | 10^-7^ M vs. 10^-3^ M | ns | ns | *** | ** | 0.9939 | 0.0687 | 0.0008 | 0.0037 |
|  | 10^-6^ M vs. 10^-5^ M | ns | ns | ns | ns | 0.5662 | 0.3756 | 0.9966 | 0.9577 |
|  | 10^-6^ M vs. 10^-4^ M | * | ns | ns | ns | 0.0117 | 0.4970 | >0.9999 | 0.5733 |
|  | 10^-6^ M vs. 10^-3^ M | ns | ns | ns | ** | 0.8371 | 0.9911 | 0.4367 | 0.0042 |
|  | 10^-5^ M vs. 10^-4^ M | ** | * | ns | ns | 0.0029 | 0.0488 | 0.9949 | 0.2554 |
|  | 10^-5^ M vs. 10^-3^M | ns | ns | ns | ** | 0.9912 | 0.6305 | 0.6517 | 0.0023 |
|  | 10^-4^ M vs. 10^-3^ M | ** | ns | ns | * | 0.0044 | 0.2818 | 0.4188 | 0.0183 |
| 40 ℃ | Control vs. 10^-7^ M | ns | ns | ** | * | 0.9914 | 0.3193 | 0.0039 | 0.0286 |
|  | Control vs. 10^-6^ M | ** | ns | * | * | 0.0047 | 0.1005 | 0.0182 | 0.0326 |
|  | Control vs. 10^-5^ M | ns | ns | ns | ns | 0.6314 | 0.9890 | 0.5290 | 0.0679 |
|  | Control vs. 10^-4^ M | ns | ** | ns | ns | 0.2202 | 0.0027 | 0.2288 | 0.0686 |
|  | Control vs. 10^-3^ M | ns | ns | ns | ns | 0.0848 | 0.6769 | 0.9893 | 0.9992 |
|  | 10^-7^ M vs. 10^-6^ M | ** | ns | ns | ns | 0.0031 | 0.8882 | 0.5321 | >0.9999 |
|  | 10^-7^ M vs. 10^-5^ M | ns | ns | * | ns | 0.3784 | 0.5660 | 0.0183 | 0.9515 |
|  | 10^-7^ M vs. 10^-4^ M | ns | *** | * | ns | 0.1195 | 0.0006 | 0.0408 | 0.9493 |
|  | 10^-7^ M vs. 10^-3^ M | * | ns | ** | * | 0.0471 | 0.0607 | 0.0062 | 0.0208 |
|  | 10^-6^ M vs. 10^-5^ M | * | ns | ns | ns | 0.0190 | 0.1920 | 0.1181 | 0.9755 |
|  | 10^-6^ M vs. 10^-4^ M | ns | *** | ns | ns | 0.0548 | 0.0003 | 0.2903 | 0.9740 |
|  | 10^-6^ M vs. 10^-3^ M | ns | * | * | * | 0.1402 | 0.0209 | 0.0319 | 0.0236 |
|  | 10^-5^ M vs. 10^-4^ M | ns | ** | ns | ns | 0.8859 | 0.0018 | 0.9557 | >0.9999 |
|  | 10^-5^ M vs. 10^-3^M | ns | ns | ns | * | 0.4784 | 0.4011 | 0.8150 | 0.0480 |
|  | 10^-4^ M vs. 10^-3^ M | ns | ** | ns | * | 0.9450 | 0.0090 | 0.4228 | 0.0485 |

*indicates *p*<0.05; **indicates *p*<0.01; ***indicates *p*<0.001; ****indicates *p*<0.0001; ns indicates not significant

**Table S9.** Two-way ANOVA results for the effects of temperature and putrescine concentration on Chl b.

| Factors | Day 2 | | Day 4 | | Day 6 | | Day 8 | |
| --- | --- | --- | --- | --- | --- | --- | --- | --- |
|  | F | *p* | F | *p* | F | *p* | F | *p* |
| Temperature | 17.407 | <0.001 | 51.721 | <0.001 | 50.207 | <0.001 | 96.425 | <0.001 |
| Putrescine | 2.631 | 0.059 | 4.554 | <0.005 | 3.964 | 0.013 | 6.295 | <0.05 |
| Temperature* Putrescine | 4.584 | <0.05 | 7.730 | <0.001 | 5.252 | <0.05 | 5.723 | <0.001 |

**Table S10.** Post-hoc comparisons of the temperature effect on Chl b within each putrescine concentration.

| Dunnett's multiple comparisons test | | Summary | | | | Adjusted *p* value | | | |
| --- | --- | --- | --- | --- | --- | --- | --- | --- | --- |
|  |  | Day 2 | Day 4 | Day 6 | Day 8 | Day 2 | Day 4 | Day 6 | Day 8 |
| 0 M | 28 ℃ vs. 34 ℃ | ns | ns | ns | * | 0.1430 | 0.2245 | >0.9999 | 0.0139 |
|  | 28 ℃ vs. 40 ℃ | ns | * | ns | * | 0.4635 | 0.0161 | 0.1308 | 0.0321 |
|  | 34 ℃ vs. 40 ℃ | ns | * | ns | ns | 0.4665 | 0.0496 | 0.1303 | 0.3245 |
| 10^-7^ M | 28 ℃ vs. 34 ℃ | ** | ns | ** | *** | 0.0089 | 0.3568 | 0.0055 | 0.0004 |
|  | 28 ℃ vs. 40 ℃ | ns | ** | * | *** | 0.5807 | 0.0029 | 0.0418 | 0.0007 |
|  | 34 ℃ vs. 40 ℃ | ** | ** | ** | ns | 0.0061 | 0.0019 | 0.0017 | 0.1581 |
| 10^-6^ M | 28 ℃ vs. 34 ℃ | * | ns | ns | * | 0.0259 | 0.7568 | 0.1698 | 0.0432 |
|  | 28 ℃ vs. 40 ℃ | ns | ns | * | * | 0.9268 | 0.1397 | 0.0281 | 0.0433 |
|  | 34 ℃ vs. 40 ℃ | * | ns | ns | ns | 0.0214 | 0.0797 | 0.1423 | >0.9999 |
| 10^-5^ M | 28 ℃ vs. 34 ℃ | ns | ns | ns | * | 0.4900 | 0.0546 | 0.4262 | 0.0301 |
|  | 28 ℃ vs. 40 ℃ | ns | * | ns | * | 0.0683 | 0.0166 | 0.1155 | 0.0280 |
|  | 34 ℃ vs. 40 ℃ | * | ns | ns | ns | 0.0313 | 0.2087 | 0.3979 | 0.9899 |
| 10^-4^ M | 28 ℃ vs. 34 ℃ | ns | ** | ** | ** | 0.0927 | 0.0069 | 0.0072 | 0.0039 |
|  | 28 ℃ vs. 40 ℃ | ns | ns | ** | ** | 0.3953 | 0.0591 | 0.0013 | 0.0055 |
|  | 34 ℃ vs. 40 ℃ | ns | * | *** | ns | 0.3294 | 0.0380 | 0.0004 | 0.5440 |
| 10^-3^ M | 28 ℃ vs. 34 ℃ | ns | ** | ns | ns | 0.9502 | 0.0051 | 0.4817 | 0.0647 |
|  | 28 ℃ vs. 40 ℃ | ** | ** | ns | * | 0.0043 | 0.0029 | 0.1356 | 0.0382 |
|  | 34 ℃ vs. 40 ℃ | ** | ns | ns | ns | 0.0048 | 0.2579 | 0.4230 | 0.7028 |

*indicates *p*<0.05; **indicates *p*<0.01; ***indicates *p*<0.001; ****indicates *p*<0.0001; ns indicates not significant.

**Table S11.** Post-hoc comparisons of the putrescine concentration effect on Chl b within each temperature.

| Dunnett's multiple comparisons test | | Summary | | | | Adjusted *p* value | | | |
| --- | --- | --- | --- | --- | --- | --- | --- | --- | --- |
|  |  | Day 2 | Day 4 | Day 6 | Day 8 | Day 2 | Day 4 | Day 6 | Day 8 |
| 28 ℃ | Control vs. 10^-7^ M | ns | ns | ns | *** | 0.8998 | 0.3298 | 0.4538 | 0.0010 |
|  | Control vs. 10^-6^ M | ns | ns | ns | ns | 0.8974 | 0.4054 | 0.8900 | 0.1271 |
|  | Control vs. 10^-5^ M | ns | ns | ns | ns | 0.8822 | 0.4013 | 0.8433 | 0.2853 |
|  | Control vs. 10^-4^ M | ns | * | ns | ns | 0.4822 | 0.0479 | 0.9332 | 0.6597 |
|  | Control vs. 10^-3^ M | * | ** | ns | ns | 0.0174 | 0.0020 | 0.8086 | 0.8589 |
|  | 10^-7^ M vs. 10^-6^ M | ns | * | ns | *** | 0.4248 | 0.0346 | 0.9275 | 0.0002 |
|  | 10^-7^ M vs. 10^-5^ M | ns | ns | ns | ** | >0.9999 | >0.9999 | 0.9571 | 0.0055 |
|  | 10^-7^ M vs. 10^-4^ M | ns | ns | ns | ** | 0.9371 | 0.5484 | 0.8826 | 0.0028 |
|  | 10^-7^ M vs. 10^-3^ M | * | * | ns | ** | 0.0477 | 0.0125 | 0.1187 | 0.0021 |
|  | 10^-6^ M vs. 10^-5^ M | ns | * | ns | * | 0.4043 | 0.0420 | >0.9999 | 0.0110 |
|  | 10^-6^ M vs. 10^-4^ M | ns | ** | ns | * | 0.1626 | 0.0068 | >0.9999 | 0.0249 |
|  | 10^-6^ M vs. 10^-3^ M | ** | *** | ns | * | 0.0070 | 0.0005 | 0.3292 | 0.0379 |
|  | 10^-5^ M vs. 10^-4^ M | ns | ns | ns | ns | 0.9493 | 0.4598 | 0.9998 | 0.9419 |
|  | 10^-5^ M vs. 10^-3^M | ns | * | ns | ns | 0.0503 | 0.0105 | 0.2896 | 0.7846 |
|  | 10^-4^ M vs. 10^-3^ M | ns | ns | ns | ns | 0.1255 | 0.0724 | 0.3796 | 0.9975 |
| 34 ℃ | Control vs. 10^-7^ M | ns | ** | ns | ns | 0.0694 | 0.0027 | 0.0820 | 0.8192 |
|  | Control vs. 10^-6^ M | ns | ns | ns | ns | 0.9998 | 0.9957 | 0.3771 | >0.9999 |
|  | Control vs. 10^-5^ M | ns | ns | ns | ns | 0.8780 | >0.9999 | 0.4323 | 0.7540 |
|  | Control vs. 10^-4^ M | * | ns | ns | ns | 0.0262 | 0.1729 | 0.9178 | 0.9887 |
|  | Control vs. 10^-3^ M | ns | ns | ns | ** | 0.5614 | 0.5585 | 0.9289 | 0.0027 |
|  | 10^-7^ M vs. 10^-6^ M | ns | ** | ** | ns | 0.0908 | 0.0039 | 0.0098 | 0.8404 |
|  | 10^-7^ M vs. 10^-5^ M | * | ** | * | ns | 0.0230 | 0.0027 | 0.0110 | 0.2403 |
|  | 10^-7^ M vs. 10^-4^ M | ** | *** | ns | ns | 0.0013 | 0.0004 | 0.2372 | 0.9842 |
|  | 10^-7^ M vs. 10^-3^ M | ns | *** | * | ** | 0.4581 | 0.0008 | 0.0312 | 0.0012 |
|  | 10^-6^ M vs. 10^-5^ M | ns | ns | ns | ns | 0.7714 | 0.9947 | >0.9999 | 0.7299 |
|  | 10^-6^ M vs. 10^-4^ M | * | ns | ns | ns | 0.0207 | 0.1022 | 0.1323 | 0.9924 |
|  | 10^-6^ M vs. 10^-3^ M | ns | ns | ns | ** | 0.6857 | 0.3524 | 0.8127 | 0.0027 |
|  | 10^-5^ M vs. 10^-4^ M | ns | ns | ns | ns | 0.0803 | 0.1770 | 0.1538 | 0.4657 |
|  | 10^-5^ M vs. 10^-3^M | ns | ns | ns | ** | 0.1843 | 0.5688 | 0.8697 | 0.0080 |
|  | 10^-4^ M vs. 10^-3^ M | ** | ns | ns | ** | 0.0055 | 0.8581 | 0.4940 | 0.0018 |
| 40 ℃ | Control vs. 10^-7^ M | ns | ns | ns | ns | 0.4409 | 0.7974 | 0.8407 | 0.9999 |
|  | Control vs. 10^-6^ M | * | ns | ns | ns | 0.0374 | 0.9167 | 0.7501 | 0.8185 |
|  | Control vs. 10^-5^ M | * | ns | ns | ns | 0.0170 | 0.9509 | 0.9514 | 0.9256 |
|  | Control vs. 10^-4^ M | ns | * | ns | ns | 0.5469 | 0.0233 | 0.9406 | 0.9726 |
|  | Control vs. 10^-3^ M | ** | ns | ns | ns | 0.0036 | 0.9965 | >0.9999 | 0.9972 |
|  | 10^-7^ M vs. 10^-6^ M | ns | ns | ns | ns | 0.3274 | 0.9995 | >0.9999 | 0.9033 |
|  | 10^-7^ M vs. 10^-5^ M | ns | ns | ns | ns | 0.1349 | 0.3980 | 0.9991 | 0.9732 |
|  | 10^-7^ M vs. 10^-4^ M | ns | ** | ns | ns | 0.9999 | 0.0074 | 0.9996 | 0.9941 |
|  | 10^-7^ M vs. 10^-3^ M | * | ns | ns | ns | 0.0195 | 0.9556 | 0.9010 | 0.9828 |
|  | 10^-6^ M vs. 10^-5^ M | ns | ns | ns | ns | 0.9572 | 0.5333 | 0.9926 | 0.9996 |
|  | 10^-6^ M vs. 10^-4^ M | ns | ** | ns | ns | 0.2558 | 0.0096 | 0.9950 | 0.9945 |
|  | 10^-6^ M vs. 10^-3^ M | ns | ns | ns | ns | 0.2199 | 0.9939 | 0.8237 | 0.6066 |
|  | 10^-5^ M vs. 10^-4^ M | ns | ns | ns | ns | 0.1046 | 0.0545 | >0.9999 | >0.9999 |
|  | 10^-5^ M vs. 10^-3^M | ns | ns | ns | ns | 0.5084 | 0.7875 | 0.9792 | 0.7512 |
|  | 10^-4^ M vs. 10^-3^ M | * | * | ns | ns | 0.0157 | 0.0152 | 0.9728 | 0.8478 |

*indicates *p*<0.05; **indicates *p*<0.01; ***indicates *p*<0.001; ****indicates *p*<0.0001; ns indicates not significant.

**Table S12.** Two-way ANOVA results for the effects of temperature and putrescine concentration on Chl a/Chl b.

| Factors | Day 2 | | Day 4 | | Day 6 | | Day 8 | |
| --- | --- | --- | --- | --- | --- | --- | --- | --- |
|  | F | *p* | F | *p* | F | *p* | F | *p* |
| Temperature | 52.534 | <0.001 | 2.032 | 0.092 | 2.478 | 0.092 | 0.87 | 0.092 |
| Putrescine | 1.088 | 0.092 | 0.996 | 0.092 | 0.827 | 0.092 | 0.708 | 0.092 |
| Temperature* Putrescine | 1.059 | 0.092 | 1.891 | 0.092 | 0.623 | 0.092 | 0.953 | 0.092 |

**Table S13.** Post-hoc comparisons of the temperature effect on Chl a/Chl b within each putrescine concentration.

| Dunnett's multiple comparisons test | | Summary | | | | Adjusted *p* value | | | |
| --- | --- | --- | --- | --- | --- | --- | --- | --- | --- |
|  |  | Day 2 | Day 4 | Day 6 | Day 8 | Day 2 | Day 4 | Day 6 | Day 8 |
| 0 M | 28 ℃ vs. 34 ℃ | ** | ns | ns | * | 0.0074 | 0.2095 | 0.5301 | 0.0474 |
|  | 28 ℃ vs. 40 ℃ | ns | ns | ns | * | 0.1960 | 0.2927 | 0.9648 | 0.0431 |
|  | 34 ℃ vs. 40 ℃ | * | ns | ns | ** | 0.0186 | 0.0524 | 0.6550 | 0.0066 |
| 10^-7^ M | 28 ℃ vs. 34 ℃ | ** | * | * | ns | 0.0036 | 0.0318 | 0.0273 | 0.2228 |
|  | 28 ℃ vs. 40 ℃ | ns | ns | ** | ** | 0.4189 | 0.6247 | 0.0043 | 0.0020 |
|  | 34 ℃ vs. 40 ℃ | ** | ns | * | ** | 0.0055 | 0.0575 | 0.0342 | 0.0013 |
| 10^-6^ M | 28 ℃ vs. 34 ℃ | ** | ** | ns | *** | 0.0023 | 0.0037 | 0.8593 | 0.0008 |
|  | 28 ℃ vs. 40 ℃ | ** | ** | * | ** | 0.0061 | 0.0026 | 0.0433 | 0.0011 |
|  | 34 ℃ vs. 40 ℃ | ns | ns | * | ns | 0.0755 | 0.4913 | 0.0319 | 0.2993 |
| 10^-5^ M | 28 ℃ vs. 34 ℃ | ** | *** | * | ** | 0.0015 | 0.0007 | 0.0461 | 0.0026 |
|  | 28 ℃ vs. 40 ℃ | ns | * | ns | ns | 0.4842 | 0.0121 | 0.9237 | 0.1212 |
|  | 34 ℃ vs. 40 ℃ | ** | ** | * | ** | 0.0020 | 0.0025 | 0.0368 | 0.0014 |
| 10^-4^ M | 28 ℃ vs. 34 ℃ | ** | ** | *** | ns | 0.0029 | 0.0033 | 0.0002 | 0.1040 |
|  | 28 ℃ vs. 40 ℃ | ns | * | *** | * | 0.4175 | 0.0233 | 0.0006 | 0.0171 |
|  | 34 ℃ vs. 40 ℃ | ** | * | * | ns | 0.0044 | 0.0245 | 0.0144 | 0.1044 |
| 10^-3^ M | 28 ℃ vs. 34 ℃ | ** | ns | **** | ns | 0.0017 | 0.0817 | <0.0001 | 0.1018 |
|  | 28 ℃ vs. 40 ℃ | ns | *** | ** | * | 0.9458 | 0.0008 | 0.0053 | 0.0292 |
|  | 34 ℃ vs. 40 ℃ | ** | *** | *** | ns | 0.0018 | 0.0005 | 0.0002 | 0.2630 |

*indicates *p*<0.05; **indicates *p*<0.01; ***indicates *p*<0.001; ****indicates *p*<0.0001; ns indicates not significant.

**Table S14.** Post-hoc comparisons of the putrescine concentration effect on Chl a/Chl b within each temperature.

| Dunnett's multiple comparisons test | | Summary | | | | Adjusted *p* value | | | |
| --- | --- | --- | --- | --- | --- | --- | --- | --- | --- |
|  |  | Day 2 | Day 4 | Day 6 | Day 8 | Day 2 | Day 4 | Day 6 | Day 8 |
| 28 ℃ | Control vs. 10^-7^ M | ns | ns | *** | * | 0.7688 | 0.9547 | 0.0003 | 0.0132 |
|  | Control vs. 10^-6^ M | ns | ** | ** | **** | 0.1482 | 0.0071 | 0.0050 | <0.0001 |
|  | Control vs. 10^-5^ M | ns | * | ** | ns | 0.9412 | 0.0248 | 0.0016 | 0.9149 |
|  | Control vs. 10^-4^ M | ** | * | *** | ns | 0.0036 | 0.0191 | 0.0006 | 0.2158 |
|  | Control vs. 10^-3^ M | ns | ns | ** | ns | 0.9934 | 0.9998 | 0.0010 | >0.9999 |
|  | 10^-7^ M vs. 10^-6^ M | * | * | * | **** | 0.0355 | 0.0146 | 0.0264 | <0.0001 |
|  | 10^-7^ M vs. 10^-5^ M | ns | ns | ns | * | 0.3587 | 0.0573 | 0.1374 | 0.0335 |
|  | 10^-7^ M vs. 10^-4^ M | * | ** | ns | ** | 0.0108 | 0.0091 | 0.7090 | 0.0016 |
|  | 10^-7^ M vs. 10^-3^ M | ns | ns | ns | * | 0.5107 | 0.8773 | 0.2807 | 0.0114 |
|  | 10^-6^ M vs. 10^-5^ M | ns | ns | ns | **** | 0.3844 | 0.7436 | 0.6539 | <0.0001 |
|  | 10^-6^ M vs. 10^-4^ M | *** | *** | ns | **** | 0.0005 | 0.0003 | 0.1218 | <0.0001 |
|  | 10^-6^ M vs. 10^-3^ M | ns | ** | ns | **** | 0.2631 | 0.0057 | 0.3588 | <0.0001 |
|  | 10^-5^ M vs. 10^-4^ M | ** | *** | ns | ns | 0.0019 | 0.0006 | 0.6161 | 0.0740 |
|  | 10^-5^ M vs. 10^-3^M | ns | * | ns | ns | 0.9988 | 0.0193 | 0.9828 | 0.8533 |
|  | 10^-4^ M vs. 10^-3^ M | ** | * | ns | ns | 0.0025 | 0.0246 | 0.9096 | 0.2602 |
| 34 ℃ | Control vs. 10^-7^ M | ns | * | * | ns | 0.9988 | 0.0345 | 0.0338 | 0.5989 |
|  | Control vs. 10^-6^ M | ns | ns | ** | ns | 0.0812 | 0.0870 | 0.0012 | 0.5853 |
|  | Control vs. 10^-5^ M | ns | ** | ** | ** | 0.4668 | 0.0031 | 0.0041 | 0.0014 |
|  | Control vs. 10^-4^ M | ns | ** | ns | ns | 0.6173 | 0.0041 | >0.9999 | 0.1941 |
|  | Control vs. 10^-3^ M | ns | ns | ns | ns | 0.9997 | 0.6454 | >0.9999 | 0.5327 |
|  | 10^-7^ M vs. 10^-6^ M | ns | ns | * | ns | 0.0552 | 0.9414 | 0.0428 | 0.1009 |
|  | 10^-7^ M vs. 10^-5^ M | ns | ns | ns | *** | 0.3238 | 0.1986 | 0.2954 | 0.0005 |
|  | 10^-7^ M vs. 10^-4^ M | ns | *** | * | ns | 0.4455 | 0.0003 | 0.0287 | 0.8670 |
|  | 10^-7^ M vs. 10^-3^ M | ns | ns | * | ns | 0.9841 | 0.1889 | 0.0327 | >0.9999 |
|  | 10^-6^ M vs. 10^-5^ M | ns | ns | ns | ** | 0.6242 | 0.0754 | 0.5461 | 0.0049 |
|  | 10^-6^ M vs. 10^-4^ M | ns | *** | ** | * | 0.4729 | 0.0004 | 0.0010 | 0.0314 |
|  | 10^-6^ M vs. 10^-3^ M | ns | ns | ** | ns | 0.1097 | 0.4765 | 0.0011 | 0.0871 |
|  | 10^-5^ M vs. 10^-4^ M | ns | **** | ** | *** | 0.9994 | <0.0001 | 0.0036 | 0.0003 |
|  | 10^-5^ M vs. 10^-3^M | ns | * | ** | *** | 0.5982 | 0.0113 | 0.0039 | 0.0005 |
|  | 10^-4^ M vs. 10^-3^ M | ns | ** | ns | ns | 0.7553 | 0.0013 | >0.9999 | 0.9144 |
| 40 ℃ | Control vs. 10^-7^ M | ns | ns | ns | **** | 0.9821 | 0.3413 | 0.0691 | <0.0001 |
|  | Control vs. 10^-6^ M | ns | *** | ns | *** | 0.3618 | 0.0008 | 0.4278 | 0.0006 |
|  | Control vs. 10^-5^ M | ns | ns | ** | ns | 0.4625 | 0.2129 | 0.0048 | 0.3429 |
|  | Control vs. 10^-4^ M | ns | ns | ns | *** | 0.7991 | >0.9999 | 0.9718 | 0.0006 |
|  | Control vs. 10^-3^ M | ns | **** | * | *** | 0.3189 | <0.0001 | 0.0244 | 0.0008 |
|  | 10^-7^ M vs. 10^-6^ M | ns | ** | ** | ** | 0.1782 | 0.0036 | 0.0095 | 0.0016 |
|  | 10^-7^ M vs. 10^-5^ M | ns | ns | *** | **** | 0.7825 | 0.9970 | 0.0004 | <0.0001 |
|  | 10^-7^ M vs. 10^-4^ M | ns | ns | * | ** | 0.4786 | 0.2869 | 0.0323 | 0.0016 |
|  | 10^-7^ M vs. 10^-3^ M | ns | **** | ** | ** | 0.6000 | <0.0001 | 0.0013 | 0.0011 |
|  | 10^-6^ M vs. 10^-5^ M | * | ** | * | ** | 0.0434 | 0.0051 | 0.0293 | 0.0024 |
|  | 10^-6^ M vs. 10^-4^ M | ns | *** | ns | ns | 0.9260 | 0.0007 | 0.7802 | >0.9999 |
|  | 10^-6^ M vs. 10^-3^ M | * | **** | ns | ns | 0.0299 | <0.0001 | 0.2134 | 0.9931 |
|  | 10^-5^ M vs. 10^-4^ M | ns | ns | ** | ** | 0.1185 | 0.1777 | 0.0087 | 0.0024 |
|  | 10^-5^ M vs. 10^-3^M | ns | **** | ns | ** | 0.9987 | <0.0001 | 0.5095 | 0.0036 |
|  | 10^-4^ M vs. 10^-3^ M | ns | **** | ns | ns | 0.0793 | <0.0001 | 0.0512 | 0.9931 |

*indicates *p*<0.05; **indicates *p*<0.01; ***indicates *p*<0.001; ****indicates *p*<0.0001; ns indicates not significant.

Table S15. Table S1. Two-way ANOVA results for the effects of temperature and putrescine concentration on P_n_, R, and P_g_.

| Factors | P_n_ | | R | | P_g_ | |
| --- | --- | --- | --- | --- | --- | --- |
|  | F | *p* | F | *p* | F | *p* |
| Temperature | 85.666 | <0.001 | 61.611 | <0.001 | 108.617 | <0.001 |
| Putrescine | 122.016 | <0.001 | 116.629 | <0.001 | 190.621 | <0.001 |
| Temperature* Putrescine | 7.970 | <0.001 | 11.654 | <0.001 | 9.743 | <0.001 |

**Table S16.** Post-hoc comparisons of the temperature effect on P_n_, R, and P_g_ within each putrescine concentration.

| Dunnett's multiple comparisons test | | Summary | | | Adjusted *p* value | | |
| --- | --- | --- | --- | --- | --- | --- | --- |
|  |  | P_n_ | R | P_g_ | P_n_ | R | P_g_ |
| 0 M | 28 ℃ vs. 34 ℃ | ns | ns | ns | 0.8330 | 0.2676 | 0.2879 |
|  | 28 ℃ vs. 40 ℃ | ns | ns | ns | 0.4115 | 0.4245 | 0.3529 |
|  | 34 ℃ vs. 40 ℃ | ns | ns | ns | 0.2413 | 0.0831 | 0.0762 |
| 10^-7^ M | 28 ℃ vs. 34 ℃ | ns | *** | ** | 0.4701 | 0.0007 | 0.0011 |
|  | 28 ℃ vs. 40 ℃ | ns | **** | **** | 0.1820 | <0.0001 | <0.0001 |
|  | 34 ℃ vs. 40 ℃ | ns | **** | *** | 0.5917 | <0.0001 | 0.0003 |
| 10^-6^ M | 28 ℃ vs. 34 ℃ | ns | ns | ns | 0.5349 | 0.1666 | 0.1167 |
|  | 28 ℃ vs. 40 ℃ | ns | ns | ns | 0.0682 | 0.1818 | 0.0622 |
|  | 34 ℃ vs. 40 ℃ | ns | ns | ns | 0.1668 | 0.9940 | 0.6865 |
| 10^-5^ M | 28 ℃ vs. 34 ℃ | ns | ns | * | 0.2355 | 0.0621 | 0.0120 |
|  | 28 ℃ vs. 40 ℃ | * | * | ** | 0.0489 | 0.0100 | 0.0017 |
|  | 34 ℃ vs. 40 ℃ | ns | ns | * | 0.2355 | 0.0701 | 0.0133 |
| 10^-4^ M | 28 ℃ vs. 34 ℃ | * | ns | ns | 0.0315 | 0.1556 | 0.4080 |
|  | 28 ℃ vs. 40 ℃ | ** | ns | ns | 0.0021 | 0.7765 | 0.0987 |
|  | 34 ℃ vs. 40 ℃ | ** | ns | * | 0.0088 | 0.0901 | 0.0373 |
| 10^-3^ M | 28 ℃ vs. 34 ℃ | ** | ns | ns | 0.0091 | 0.0797 | 0.2291 |
|  | 28 ℃ vs. 40 ℃ | ** | ns | * | 0.0014 | 0.2545 | 0.0248 |
|  | 34 ℃ vs. 40 ℃ | * | * | ** | 0.0125 | 0.0241 | 0.0098 |

*indicates *p*<0.05; **indicates *p*<0.01; ***indicates *p*<0.001; ****indicates *p*<0.0001; ns indicates not significant.

**Table S17.** Two-way ANOVA results for the effects of temperature and putrescine concentration on Fv/Fm.

| Factors | Day 2 | | Day 4 | | Day 6 | | Day 8 | |
| --- | --- | --- | --- | --- | --- | --- | --- | --- |
|  | F | *p* | F | *p* | F | *p* | F | *p* |
| Temperature | 40.657 | <0.001 | 880.209 | <0.001 | 2214.068 | <0.001 | 87.631 | <0.001 |
| Putrescine | 45.869 | <0.001 | 58.444 | <0.001 | 251.444 | <0.001 | 4.674 | <0.05 |
| Temperature* Putrescine | 17.903 | <0.001 | 19.276 | <0.001 | 132.600 | <0.001 | 2.860 | 0.025 |

**Table S18.** Post-hoc comparisons of the temperature effect on Fv/Fm within each putrescine concentration.

| Dunnett's multiple comparisons test | | Summary | | | | Adjusted *p* value | | | |
| --- | --- | --- | --- | --- | --- | --- | --- | --- | --- |
|  |  | Day 2 | Day 4 | Day 6 | Day 8 | Day 2 | Day 4 | Day 6 | Day 8 |
| 0 M | 28 ℃ vs. 34 ℃ | * | ** | ns | * | 0.0194 | 0.0091 | 0.1259 | 0.0444 |
|  | 28 ℃ vs. 40 ℃ | * | *** | *** | ** | 0.0169 | 0.0002 | 0.0002 | 0.0033 |
|  | 34 ℃ vs. 40 ℃ | ns | *** | *** | * | 0.9533 | 0.0008 | 0.0003 | 0.0141 |
| 10^-7^ M | 28 ℃ vs. 34 ℃ | * | * | ns | ns | 0.0288 | 0.0216 | 0.0923 | 0.9874 |
|  | 28 ℃ vs. 40 ℃ | ns | *** | *** | ** | 0.6798 | 0.0004 | 0.0006 | 0.0032 |
|  | 34 ℃ vs. 40 ℃ | * | *** | *** | ** | 0.0479 | 0.0009 | 0.0004 | 0.0031 |
| 10^-6^ M | 28 ℃ vs. 34 ℃ | * | ** | ns | ns | 0.0284 | 0.0014 | 0.1951 | 0.1127 |
|  | 28 ℃ vs. 40 ℃ | ns | **** | *** | ** | 0.4146 | <0.0001 | 0.0005 | 0.0017 |
|  | 34 ℃ vs. 40 ℃ | * | **** | *** | ** | 0.0141 | <0.0001 | 0.0003 | 0.0033 |
| 10^-5^ M | 28 ℃ vs. 34 ℃ | * | ns | * | ** | 0.0178 | 0.0607 | 0.0323 | 0.0070 |
|  | 28 ℃ vs. 40 ℃ | ns | ** | **** | ** | >0.9999 | 0.0038 | <0.0001 | 0.0014 |
|  | 34 ℃ vs. 40 ℃ | * | * | *** | * | 0.0178 | 0.0145 | 0.0001 | 0.0171 |
| 10^-4^ M | 28 ℃ vs. 34 ℃ | * | *** | *** | ** | 0.0116 | 0.0006 | 0.0006 | 0.0046 |
|  | 28 ℃ vs. 40 ℃ | ns | **** | **** | ** | 0.0623 | <0.0001 | <0.0001 | 0.0018 |
|  | 34 ℃ vs. 40 ℃ | ** | *** | **** | ns | 0.0034 | 0.0010 | <0.0001 | 0.0637 |
| 10^-3^ M | 28 ℃ vs. 34 ℃ | ns | ** | **** | ns | 0.0717 | 0.0042 | <0.0001 | 0.2920 |
|  | 28 ℃ vs. 40 ℃ | ns | ** | *** | ns | 0.4944 | 0.0056 | 0.0004 | 0.2257 |
|  | 34 ℃ vs. 40 ℃ | * | ns | ** | ns | 0.0328 | 0.6480 | 0.0050 | 0.9553 |

*indicates *p*<0.05; **indicates *p*<0.01; ***indicates *p*<0.001; ****indicates *p*<0.0001; ns indicates not significant.

**Table S19.** Post-hoc comparisons of the putrescine concentration effect on Fv/Fm within each temperature.

| Dunnett's multiple comparisons test | | Summary | | | | Adjusted *p* value | | | |
| --- | --- | --- | --- | --- | --- | --- | --- | --- | --- |
|  |  | Day 2 | Day 4 | Day 6 | Day 8 | Day 2 | Day 4 | Day 6 | Day 8 |
| 28 ℃ | Control vs. 10^-7^ M | ns | ns | ns | ns | 0.7777 | >0.9999 | 0.5594 | 0.9826 |
|  | Control vs. 10^-6^ M | ns | ns | ** | ns | 0.9421 | 0.7209 | 0.0035 | 0.7042 |
|  | Control vs. 10^-5^ M | ns | ns | ns | ns | 0.9999 | 0.9741 | 0.9421 | 0.7837 |
|  | Control vs. 10^-4^ M | ns | ns | ns | ns | 0.3670 | 0.7209 | >0.9999 | 0.6219 |
|  | Control vs. 10^-3^ M | ** | ** | **** | ns | 0.0029 | 0.0054 | <0.0001 | 0.8552 |
|  | 10^-7^ M vs. 10^-6^ M | ns | ns | * | ns | 0.3670 | 0.7209 | 0.0149 | 0.9565 |
|  | 10^-7^ M vs. 10^-5^ M | ns | ns | ns | ns | 0.8724 | 0.9741 | 0.2298 | 0.9826 |
|  | 10^-7^ M vs. 10^-4^ M | ns | ns | ns | ns | 0.0874 | 0.7209 | 0.5594 | 0.9139 |
|  | 10^-7^ M vs. 10^-3^ M | ** | ** | **** | ns | 0.0012 | 0.0054 | <0.0001 | 0.5412 |
|  | 10^-6^ M vs. 10^-5^ M | ns | ns | ** | ns | 0.8724 | 0.3841 | 0.0018 | >0.9999 |
|  | 10^-6^ M vs. 10^-4^ M | ns | ns | ** | ns | 0.7777 | 0.1777 | 0.0035 | >0.9999 |
|  | 10^-6^ M vs. 10^-3^ M | ** | * | *** | ns | 0.0058 | 0.0188 | 0.0006 | 0.2362 |
|  | 10^-5^ M vs. 10^-4^ M | ns | ns | ns | ns | 0.2915 | 0.9741 | 0.9421 | 0.9993 |
|  | 10^-5^ M vs. 10^-3^M | ** | ** | **** | ns | 0.0025 | 0.0031 | <0.0001 | 0.2819 |
|  | 10^-4^ M vs. 10^-3^ M | * | ** | **** | ns | 0.0183 | 0.0019 | <0.0001 | 0.1975 |
| 34 ℃ | Control vs. 10^-7^ M | ns | ns | ** | ns | 0.9477 | 0.4623 | 0.0047 | 0.5627 |
|  | Control vs. 10^-6^ M | ns | ns | ns | ns | 0.9903 | 0.9624 | 0.4167 | 0.7937 |
|  | Control vs. 10^-5^ M | ns | ns | ns | ns | 0.9996 | 0.9982 | 0.9977 | 0.9979 |
|  | Control vs. 10^-4^ M | ns | * | * | ns | 0.9477 | 0.0121 | 0.0449 | 0.9933 |
|  | Control vs. 10^-3^ M | ns | **** | **** | ns | 0.8560 | <0.0001 | <0.0001 | 0.6502 |
|  | 10^-7^ M vs. 10^-6^ M | ns | ns | * | ns | 0.9996 | 0.8401 | 0.0295 | 0.9961 |
|  | 10^-7^ M vs. 10^-5^ M | ns | ns | ** | ns | 0.9903 | 0.6521 | 0.0034 | 0.7663 |
|  | 10^-7^ M vs. 10^-4^ M | ns | ** | *** | ns | 0.5825 | 0.0025 | 0.0003 | 0.3382 |
|  | 10^-7^ M vs. 10^-3^ M | ns | **** | **** | ns | 0.9996 | <0.0001 | <0.0001 | 0.1073 |
|  | 10^-6^ M vs. 10^-5^ M | ns | ns | ns | ns | 0.9996 | 0.9982 | 0.2707 | 0.9429 |
|  | 10^-6^ M vs. 10^-4^ M | ns | ** | ** | ns | 0.7256 | 0.0062 | 0.0066 | 0.5344 |
|  | 10^-6^ M vs. 10^-3^ M | ns | **** | **** | ns | 0.9903 | <0.0001 | <0.0001 | 0.1797 |
|  | 10^-5^ M vs. 10^-4^ M | ns | ** | ns | ns | 0.8560 | 0.0086 | 0.0694 | 0.9274 |
|  | 10^-5^ M vs. 10^-3^M | ns | **** | **** | ns | 0.9477 | <0.0001 | <0.0001 | 0.4539 |
|  | 10^-4^ M vs. 10^-3^ M | ns | **** | **** | ns | 0.4488 | <0.0001 | <0.0001 | 0.8901 |
| 40 ℃ | Control vs. 10^-7^ M | *** | ns | ns | ns | 0.0001 | 0.6742 | 0.9813 | 0.5083 |
|  | Control vs. 10^-6^ M | *** | ns | ns | * | 0.0002 | >0.9999 | 0.9813 | 0.0197 |
|  | Control vs. 10^-5^ M | *** | ns | ns | ** | 0.0002 | 0.2259 | 0.4507 | 0.0016 |
|  | Control vs. 10^-4^ M | *** | ns | ns | ns | 0.0005 | >0.9999 | 0.8607 | 0.0694 |
|  | Control vs. 10^-3^ M | *** | ns | ns | ns | 0.0005 | 0.9949 | 0.7732 | 0.8124 |
|  | 10^-7^ M vs. 10^-6^ M | ns | ns | ns | ** | 0.8674 | 0.6968 | 0.7732 | 0.0040 |
|  | 10^-7^ M vs. 10^-5^ M | ns | ns | ns | *** | 0.9913 | 0.8626 | 0.2251 | 0.0005 |
|  | 10^-7^ M vs. 10^-4^ M | ns | ns | ns | * | 0.1965 | 0.6515 | 0.5425 | 0.0112 |
|  | 10^-7^ M vs. 10^-3^ M | **** | ns | ns | ns | <0.0001 | 0.4372 | 0.9813 | 0.1367 |
|  | 10^-6^ M vs. 10^-5^ M | ns | ns | ns | ns | 0.9913 | 0.2373 | 0.7732 | 0.1367 |
|  | 10^-6^ M vs. 10^-4^ M | ns | ns | ns | ns | 0.6040 | >0.9999 | 0.9961 | 0.8124 |
|  | 10^-6^ M vs. 10^-3^ M | **** | ns | ns | ns | <0.0001 | 0.9924 | 0.4507 | 0.0694 |
|  | 10^-5^ M vs. 10^-4^ M | ns | ns | ns | * | 0.3573 | 0.2150 | 0.9459 | 0.0363 |
|  | 10^-5^ M vs. 10^-3^M | **** | ns | ns | ** | <0.0001 | 0.1309 | 0.1082 | 0.0040 |
|  | 10^-4^ M vs. 10^-3^ M | **** | ns | ns | ns | <0.0001 | 0.9967 | 0.2794 | 0.2707 |

*indicates *p*<0.05; **indicates *p*<0.01; ***indicates *p*<0.001; ****indicates *p*<0.0001; ns indicates not significant.

**Table S20.** Two-way ANOVA results for the effects of temperature and putrescine concentration on Y(II).

| Factors | Day 2 | | Day 4 | | Day 6 | | Day 8 | |
| --- | --- | --- | --- | --- | --- | --- | --- | --- |
|  | F | *p* | F | *p* | F | *p* | F | *p* |
| Temperature | 15.278 | <0.001 | 333.997 | <0.001 | 78.593 | <0.001 | 228.285 | <0.001 |
| Putrescine | 7.490 | <0.001 | 8.117 | <0.001 | 4.176 | 0.011 | 11.959 | <0.001 |
| Temperature* Putrescine | 0.713 | 0.092 | 4.079 | <0.05 | 1.602 | 0.092 | 7.429 | <0.001 |

**Table S21.** Post-hoc comparisons of the temperature effect on Y(II) within each putrescine concentration.

| Dunnett's multiple comparisons test | | Summary | | | | Adjusted *p* value | | | |
| --- | --- | --- | --- | --- | --- | --- | --- | --- | --- |
|  |  | Day 2 | Day 4 | Day 6 | Day 8 | Day 2 | Day 4 | Day 6 | Day 8 |
| 0 M | 28 ℃ vs. 34 ℃ | ns | ns | ns | ns | 0.8032 | 0.8032 | 0.4061 | >0.9999 |
|  | 28 ℃ vs. 40 ℃ | ns | ** | ** | ** | 0.3589 | 0.0019 | 0.0033 | 0.0054 |
|  | 34 ℃ vs. 40 ℃ | ns | ** | ** | ** | 0.2006 | 0.0022 | 0.0051 | 0.0054 |
| 10^-7^ M | 28 ℃ vs. 34 ℃ | ns | ns | ns | ns | 0.6394 | 0.1550 | 0.9373 | 0.3447 |
|  | 28 ℃ vs. 40 ℃ | ns | *** | * | ** | 0.3140 | 0.0006 | 0.0135 | 0.0094 |
|  | 34 ℃ vs. 40 ℃ | ns | *** | * | ** | 0.1363 | 0.0009 | 0.0117 | 0.0053 |
| 10^-6^ M | 28 ℃ vs. 34 ℃ | ** | ** | ns | ns | 0.0064 | 0.0053 | 0.2588 | 0.5185 |
|  | 28 ℃ vs. 40 ℃ | ns | *** | *** | ** | 0.3568 | 0.0002 | 0.0009 | 0.0016 |
|  | 34 ℃ vs. 40 ℃ | * | *** | ** | ** | 0.0116 | 0.0009 | 0.0012 | 0.0020 |
| 10^-5^ M | 28 ℃ vs. 34 ℃ | ns | ns | * | ns | 0.0853 | 0.0827 | 0.0460 | 0.8851 |
|  | 28 ℃ vs. 40 ℃ | ns | ** | *** | ** | 0.9786 | 0.0039 | 0.0005 | 0.0031 |
|  | 34 ℃ vs. 40 ℃ | ns | * | *** | ** | 0.0985 | 0.0122 | 0.0009 | 0.0027 |
| 10^-4^ M | 28 ℃ vs. 34 ℃ | ns | ns | ** | ns | 0.6755 | 0.6900 | 0.0041 | 0.0802 |
|  | 28 ℃ vs. 40 ℃ | ns | * | ** | * | 0.3627 | 0.0114 | 0.0010 | 0.0280 |
|  | 34 ℃ vs. 40 ℃ | ns | * | * | ns | 0.1643 | 0.0165 | 0.0149 | 0.3292 |
| 10^-3^ M | 28 ℃ vs. 34 ℃ | ** | *** | ns | ns | 0.0020 | 0.0008 | 0.5620 | 0.9690 |
|  | 28 ℃ vs. 40 ℃ | ns | **** | ns | * | 0.3292 | <0.0001 | 0.2545 | 0.0458 |
|  | 34 ℃ vs. 40 ℃ | ** | ** | ns | * | 0.0014 | 0.0012 | 0.6830 | 0.0397 |

*indicates *p*<0.05; **indicates *p*<0.01; ***indicates *p*<0.001; ****indicates *p*<0.0001; ns indicates not significant.

**Table S22.** Post-hoc comparisons of the putrescine concentration effect on Y(II) within each temperature.

| Dunnett's multiple comparisons test | | Summary | | | | Adjusted *p* value | | | |
| --- | --- | --- | --- | --- | --- | --- | --- | --- | --- |
|  |  | Day 2 | Day 4 | Day 6 | Day 8 | Day 2 | Day 4 | Day 6 | Day 8 |
| 28 ℃ | Control vs. 10^-7^ M | ns | ns | ns | ns | 0.9913 | 0.1266 | 0.9768 | 0.9998 |
|  | Control vs. 10^-6^ M | ns | ns | ns | ns | 0.7877 | 0.1266 | 0.9999 | 0.3683 |
|  | Control vs. 10^-5^ M | ns | * | ns | ns | 0.9913 | 0.0127 | 0.9768 | 0.5847 |
|  | Control vs. 10^-4^ M | ns | ns | ns | ns | 0.9295 | 0.0771 | 0.9999 | 0.7073 |
|  | Control vs. 10^-3^ M | ns | ns | ns | ns | 0.0711 | 0.1266 | 0.0544 | 0.0750 |
|  | 10^-7^ M vs. 10^-6^ M | ns | ns | ns | ns | 0.5137 | >0.9999 | 0.9961 | 0.2851 |
|  | 10^-7^ M vs. 10^-5^ M | ns | ns | ns | ns | 0.8674 | 0.3407 | 0.7398 | 0.4691 |
|  | 10^-7^ M vs. 10^-4^ M | ns | ns | ns | ns | 0.9987 | 0.9965 | 0.9282 | 0.5847 |
|  | 10^-7^ M vs. 10^-3^ M | * | ** | ns | ns | 0.0397 | 0.0059 | 0.1156 | 0.0977 |
|  | 10^-6^ M vs. 10^-5^ M | ns | ns | ns | ns | 0.9704 | 0.3407 | 0.9282 | 0.9952 |
|  | 10^-6^ M vs. 10^-4^ M | ns | ns | ns | ns | 0.3573 | 0.9965 | 0.9961 | 0.9720 |
|  | 10^-6^ M vs. 10^-3^ M | ns | ** | ns | ** | 0.2940 | 0.0059 | 0.0697 | 0.0089 |
|  | 10^-5^ M vs. 10^-4^ M | ns | ns | ns | ns | 0.6974 | 0.5315 | 0.9961 | 0.9998 |
|  | 10^-5^ M vs. 10^-3^M | ns | ** | * | * | 0.1304 | 0.0011 | 0.0268 | 0.0137 |
|  | 10^-4^ M vs. 10^-3^ M | * | ** | * | * | 0.0274 | 0.0041 | 0.0427 | 0.0172 |
| 34 ℃ | Control vs. 10^-7^ M | ns | ns | ns | ns | 0.1266 | 0.9913 | 0.9914 | 0.6852 |
|  | Control vs. 10^-6^ M | ns | ns | ns | ns | 0.1266 | 0.7877 | >0.9999 | 0.6852 |
|  | Control vs. 10^-5^ M | * | ns | ns | ns | 0.0127 | 0.9913 | >0.9999 | 0.3440 |
|  | Control vs. 10^-4^ M | ns | ns | ns | ns | 0.0771 | 0.9295 | 0.4483 | 0.2634 |
|  | Control vs. 10^-3^ M | ns | ns | ns | ns | 0.1266 | 0.0711 | 0.1977 | 0.0874 |
|  | 10^-7^ M vs. 10^-6^ M | ns | ns | ns | ns | >0.9999 | 0.5137 | 0.9969 | >0.9999 |
|  | 10^-7^ M vs. 10^-5^ M | ns | ns | ns | ns | 0.3407 | 0.8674 | 0.9969 | 0.9683 |
|  | 10^-7^ M vs. 10^-4^ M | ns | ns | ns | ns | 0.9965 | 0.9987 | 0.2520 | 0.0511 |
|  | 10^-7^ M vs. 10^-3^ M | ** | * | ns | * | 0.0059 | 0.0397 | 0.1072 | 0.0188 |
|  | 10^-6^ M vs. 10^-5^ M | ns | ns | ns | ns | 0.3407 | 0.9704 | >0.9999 | 0.9683 |
|  | 10^-6^ M vs. 10^-4^ M | ns | ns | ns | ns | 0.9965 | 0.3573 | 0.4018 | 0.0511 |
|  | 10^-6^ M vs. 10^-3^ M | ** | ns | ns | * | 0.0059 | 0.2940 | 0.1749 | 0.0188 |
|  | 10^-5^ M vs. 10^-4^ M | ns | ns | ns | * | 0.5315 | 0.6974 | 0.4018 | 0.0239 |
|  | 10^-5^ M vs. 10^-3^M | ** | ns | ns | ** | 0.0011 | 0.1304 | 0.1749 | 0.0095 |
|  | 10^-4^ M vs. 10^-3^ M | ** | * | ns | ns | 0.0041 | 0.0274 | 0.9642 | 0.9058 |
| 40 ℃ | Control vs. 10^-7^ M | ns | ns | ns | ns | 0.9768 | 0.8256 | 0.9432 | 0.9996 |
|  | Control vs. 10^-6^ M | ns | ns | ns | ns | 0.9999 | 0.6497 | 0.0890 | 0.3364 |
|  | Control vs. 10^-5^ M | ns | ns | ns | ns | 0.9768 | 0.9725 | 0.1585 | 0.5825 |
|  | Control vs. 10^-4^ M | ns | * | ns | * | 0.9999 | 0.0221 | 0.5636 | 0.0294 |
|  | Control vs. 10^-3^ M | ns | ns | ns | ns | 0.0544 | 0.0764 | 0.9432 | 0.9996 |
|  | 10^-7^ M vs. 10^-6^ M | ns | ns | ns | ns | 0.9282 | 0.9988 | 0.2331 | 0.4488 |
|  | 10^-7^ M vs. 10^-5^ M | ns | ns | ns | ns | 0.7398 | 0.9953 | 0.4055 | 0.4488 |
|  | 10^-7^ M vs. 10^-4^ M | ns | ns | ns | * | 0.9282 | 0.0764 | 0.9432 | 0.0391 |
|  | 10^-7^ M vs. 10^-3^ M | ns | ns | ns | ns | 0.1156 | 0.2887 | >0.9999 | >0.9999 |
|  | 10^-6^ M vs. 10^-5^ M | ns | ns | ns | ns | 0.9961 | 0.9492 | 0.9933 | 0.0525 |
|  | 10^-6^ M vs. 10^-4^ M | ns | ns | ns | ns | >0.9999 | 0.1136 | 0.5636 | 0.3364 |
|  | 10^-6^ M vs. 10^-3^ M | * | ns | ns | ns | 0.0427 | 0.4207 | 0.2331 | 0.4488 |
|  | 10^-5^ M vs. 10^-4^ M | ns | * | ns | ** | 0.9961 | 0.0458 | 0.8206 | 0.0063 |
|  | 10^-5^ M vs. 10^-3^M | * | ns | ns | ns | 0.0268 | 0.1698 | 0.4055 | 0.4488 |
|  | 10^-4^ M vs. 10^-3^ M | * | ns | ns | * | 0.0427 | 0.8256 | 0.9432 | 0.0391 |

*indicates *p*<0.05; **indicates *p*<0.01; ***indicates *p*<0.001; ****indicates *p*<0.0001; ns indicates not significant.

**Table S23.** Two-way ANOVA results for the effects of temperature and putrescine concentration on NPQ.

| Factors | Day 2 | | Day 4 | | Day 6 | | Day 8 | |
| --- | --- | --- | --- | --- | --- | --- | --- | --- |
|  | F | *p* | F | *p* | F | *p* | F | *p* |
| Temperature | 76.204 | <0.001 | 15.716 | <0.001 | 108.097 | <0.001 | 86.553 | <0.001 |
| Putrescine | 4.916 | <0.05 | 22.885 | <0.001 | 6.222 | <0.001 | 15.275 | <0.001 |
| Temperature* Putrescine | 2.727 | <0.05 | 14.421 | <0.001 | 2.855 | <0.05 | 8.626 | <0.001 |

**Table S24.** Post-hoc comparisons of the temperature effect on NPQ within each putrescine concentration.

| Dunnett's multiple comparisons test | | Summary | | | | Adjusted *p* value | | | |
| --- | --- | --- | --- | --- | --- | --- | --- | --- | --- |
|  |  | Day 2 | Day 4 | Day 6 | Day 8 | Day 2 | Day 4 | Day 6 | Day 8 |
| 0 M | 28 ℃ vs. 34 ℃ | ** | ns | ** | ns | 0.0076 | 0.0589 | 0.0021 | 0.1550 |
|  | 28 ℃ vs. 40 ℃ | ** | ns | ** | * | 0.0023 | 0.0589 | 0.0018 | 0.0183 |
|  | 34 ℃ vs. 40 ℃ | * | ns | ns | ns | 0.0471 | >0.9999 | 0.8264 | 0.0802 |
| 10^-7^ M | 28 ℃ vs. 34 ℃ | ns | ** | ** | ** | 0.1716 | 0.0065 | 0.0052 | 0.0073 |
|  | 28 ℃ vs. 40 ℃ | * | ** | ** | ** | 0.0286 | 0.0060 | 0.0075 | 0.0065 |
|  | 34 ℃ vs. 40 ℃ | ns | ns | ns | ns | 0.1448 | 0.9724 | 0.5604 | 0.9300 |
| 10^-6^ M | 28 ℃ vs. 34 ℃ | * | ns | *** | ns | 0.0142 | 0.6632 | 0.0004 | 0.1375 |
|  | 28 ℃ vs. 40 ℃ | ** | ns | ** | ** | 0.0020 | 0.1054 | 0.0012 | 0.0062 |
|  | 34 ℃ vs. 40 ℃ | * | ns | * | * | 0.0155 | 0.0554 | 0.0103 | 0.0180 |
| 10^-5^ M | 28 ℃ vs. 34 ℃ | * | ** | * | ns | 0.0272 | 0.0045 | 0.0138 | 0.1312 |
|  | 28 ℃ vs. 40 ℃ | * | * | * | * | 0.0139 | 0.0158 | 0.0478 | 0.0196 |
|  | 34 ℃ vs. 40 ℃ | ns | ns | ns | ** | 0.4292 | 0.0761 | 0.1754 | 0.0065 |
| 10^-4^ M | 28 ℃ vs. 34 ℃ | ns | ns | ns | ns | 0.3334 | 0.2526 | 0.1178 | 0.2852 |
|  | 28 ℃ vs. 40 ℃ | ns | ns | ns | ns | 0.1292 | 0.6004 | 0.3998 | 0.5562 |
|  | 34 ℃ vs. 40 ℃ | ns | ns | ns | ns | 0.5735 | 0.6379 | 0.4339 | 0.1098 |
| 10^-3^ M | 28 ℃ vs. 34 ℃ | * | ns | * | ns | 0.0211 | 0.9765 | 0.0283 | 0.1975 |
|  | 28 ℃ vs. 40 ℃ | ** | ns | ** | ** | 0.0086 | 0.1302 | 0.0071 | 0.0016 |
|  | 34 ℃ vs. 40 ℃ | ** | ns | ns | ** | 0.0018 | 0.1107 | 0.0906 | 0.0011 |

*indicates *p*<0.05; **indicates *p*<0.01; ***indicates *p*<0.001; ****indicates *p*<0.0001; ns indicates not significant.

**Table S25.** Post-hoc comparisons of the putrescine concentration effect on NPQ within each temperature.

| Dunnett's multiple comparisons test | | Summary | | | | Adjusted *p* value | | | |
| --- | --- | --- | --- | --- | --- | --- | --- | --- | --- |
|  |  | Day 2 | Day 4 | Day 6 | Day 8 | Day 2 | Day 4 | Day 6 | Day 8 |
| 28 ℃ | Control vs. 10^-7^ M | ns | ns | ns | ns | 0.8740 | 0.1284 | >0.9999 | 0.5955 |
|  | Control vs. 10^-6^ M | ns | ns | ns | ns | 0.9821 | 0.1427 | 0.3994 | 0.9715 |
|  | Control vs. 10^-5^ M | ns | *** | ns | ns | 0.9821 | 0.0007 | 0.9912 | 0.9457 |
|  | Control vs. 10^-4^ M | ns | ** | ns | ns | 0.9661 | 0.0020 | 0.9676 | 0.9983 |
|  | Control vs. 10^-3^ M | ns | ns | ns | ns | 0.2061 | 0.8886 | 0.8805 | 0.5955 |
|  | 10^-7^ M vs. 10^-6^ M | ns | ** | ns | ns | 0.5627 | 0.0065 | 0.4851 | 0.9227 |
|  | 10^-7^ M vs. 10^-5^ M | ns | *** | ns | ns | 0.5627 | 0.0001 | 0.9989 | 0.9555 |
|  | 10^-7^ M vs. 10^-4^ M | ns | *** | ns | ns | 0.5102 | 0.0003 | 0.9912 | 0.4160 |
|  | 10^-7^ M vs. 10^-3^ M | ns | * | ns | ns | 0.0623 | 0.0415 | 0.7972 | 0.1025 |
|  | 10^-6^ M vs. 10^-5^ M | ns | ** | ns | ns | >0.9999 | 0.0055 | 0.6604 | >0.9999 |
|  | 10^-6^ M vs. 10^-4^ M | ns | * | ns | ns | >0.9999 | 0.0257 | 0.7596 | 0.8629 |
|  | 10^-6^ M vs. 10^-3^ M | ns | ns | ns | ns | 0.4136 | 0.4411 | 0.1247 | 0.2908 |
|  | 10^-5^ M vs. 10^-4^ M | ns | ns | ns | ns | >0.9999 | 0.5749 | >0.9999 | 0.8080 |
|  | 10^-5^ M vs. 10^-3^M | ns | ** | ns | ns | 0.4136 | 0.0013 | 0.6202 | 0.2529 |
|  | 10^-4^ M vs. 10^-3^ M | ns | ** | ns | ns | 0.4603 | 0.0044 | 0.5222 | 0.7882 |
| 34 ℃ | Control vs. 10^-7^ M | ns | ns | ns | ns | 0.8219 | 0.9622 | 0.8740 | 0.1284 |
|  | Control vs. 10^-6^ M | ns | ns | ns | ns | 0.8507 | 0.9124 | 0.9821 | 0.1427 |
|  | Control vs. 10^-5^ M | ns | ns | ns | *** | 0.6579 | 0.9982 | 0.9821 | 0.0007 |
|  | Control vs. 10^-4^ M | ns | ns | ns | ** | 0.9794 | 0.7170 | 0.9661 | 0.0020 |
|  | Control vs. 10^-3^ M | ns | ns | ns | ns | 0.0692 | 0.6841 | 0.2061 | 0.8886 |
|  | 10^-7^ M vs. 10^-6^ M | ns | ns | ns | ** | 0.3048 | 0.5528 | 0.5627 | 0.0065 |
|  | 10^-7^ M vs. 10^-5^ M | ns | ns | ns | *** | 0.1958 | 0.8394 | 0.5627 | 0.0001 |
|  | 10^-7^ M vs. 10^-4^ M | ns | ns | ns | *** | 0.4918 | 0.9822 | 0.5102 | 0.0003 |
|  | 10^-7^ M vs. 10^-3^ M | ns | ns | ns | * | 0.2635 | 0.3313 | 0.0623 | 0.0415 |
|  | 10^-6^ M vs. 10^-5^ M | ns | ns | ns | ** | 0.9979 | 0.9887 | >0.9999 | 0.0055 |
|  | 10^-6^ M vs. 10^-4^ M | ns | ns | ns | * | 0.9957 | 0.2883 | >0.9999 | 0.0257 |
|  | 10^-6^ M vs. 10^-3^ M | * | ns | ns | ns | 0.0214 | 0.9933 | 0.4136 | 0.4411 |
|  | 10^-5^ M vs. 10^-4^ M | ns | ns | ns | ns | 0.9413 | 0.5213 | >0.9999 | 0.5749 |
|  | 10^-5^ M vs. 10^-3^M | * | ns | ns | ** | 0.0146 | 0.8660 | 0.4136 | 0.0013 |
|  | 10^-4^ M vs. 10^-3^ M | * | ns | ns | ** | 0.0342 | 0.1623 | 0.4603 | 0.0044 |
| 40 ℃ | Control vs. 10^-7^ M | ns | ns | ns | ns | >0.9999 | 0.0614 | 0.7570 | 0.0618 |
|  | Control vs. 10^-6^ M | ns | **** | *** | ** | 0.3994 | <0.0001 | 0.0002 | 0.0081 |
|  | Control vs. 10^-5^ M | ns | * | *** | ** | 0.9912 | 0.0123 | 0.0005 | 0.0012 |
|  | Control vs. 10^-4^ M | ns | *** | *** | *** | 0.9676 | 0.0006 | 0.0001 | 0.0001 |
|  | Control vs. 10^-3^ M | ns | ns | ns | ns | 0.8805 | 0.6143 | 0.5315 | 0.3924 |
|  | 10^-7^ M vs. 10^-6^ M | ns | *** | *** | ns | 0.4851 | 0.0005 | 0.0004 | 0.3924 |
|  | 10^-7^ M vs. 10^-5^ M | ns | ns | ** | * | 0.9989 | 0.6143 | 0.0011 | 0.0259 |
|  | 10^-7^ M vs. 10^-4^ M | ns | ** | *** | *** | 0.9912 | 0.0096 | 0.0002 | 0.0009 |
|  | 10^-7^ M vs. 10^-3^ M | ns | ns | ns | ns | 0.7972 | 0.3674 | 0.9965 | 0.5852 |
|  | 10^-6^ M vs. 10^-5^ M | ns | ** | ns | ns | 0.6604 | 0.0015 | 0.5315 | 0.2504 |
|  | 10^-6^ M vs. 10^-4^ M | ns | * | ns | ** | 0.7596 | 0.0461 | 0.7570 | 0.0041 |
|  | 10^-6^ M vs. 10^-3^ M | ns | *** | *** | ns | 0.1247 | 0.0002 | 0.0005 | 0.0618 |
|  | 10^-5^ M vs. 10^-4^ M | ns | * | ns | * | >0.9999 | 0.0461 | 0.1266 | 0.0397 |
|  | 10^-5^ M vs. 10^-3^M | ns | ns | ** | ** | 0.6202 | 0.0614 | 0.0015 | 0.0057 |
|  | 10^-4^ M vs. 10^-3^ M | ns | ** | *** | *** | 0.5222 | 0.0018 | 0.0002 | 0.0004 |

*indicates *p*<0.05; **indicates *p*<0.01; ***indicates *p*<0.001; ****indicates *p*<0.0001; ns indicates not significant.

**Table S26.** Two-way ANOVA results for the effects of temperature and putrescine concentration on φ_EO_.

| Factors | Day 2 | | Day 4 | | Day 6 | | Day 8 | |
| --- | --- | --- | --- | --- | --- | --- | --- | --- |
|  | F | *p* | F | *p* | F | *p* | F | *p* |
| Temperature | 125.913 | <0.001 | 3145.342 | <0.001 | 1878.491 | <0.001 | 1887.359 | <0.001 |
| Putrescine | 59.136 | <0.001 | 158.589 | <0.001 | 75.476 | <0.001 | 62.306 | <0.001 |
| Temperature* Putrescine | 12.915 | <0.001 | 30.679 | <0.001 | 62.167 | <0.001 | 28.364 | <0.001 |

**Table S27.** Post-hoc comparisons of the temperature effect on φ_EO_ within each putrescine concentration.

| Dunnett's multiple comparisons test | | Summary | | | | Adjusted *p* value | | | |
| --- | --- | --- | --- | --- | --- | --- | --- | --- | --- |
|  |  | Day 2 | Day 4 | Day 6 | Day 8 | Day 2 | Day 4 | Day 6 | Day 8 |
| 0 M | 28 ℃ vs. 34 ℃ | ns | ns | ns | ** | 0.1431 | 0.3413 | 0.0979 | 0.0022 |
|  | 28 ℃ vs. 40 ℃ | ns | **** | *** | *** | 0.3490 | <0.0001 | 0.0002 | 0.0003 |
|  | 34 ℃ vs. 40 ℃ | * | **** | *** | ** | 0.0446 | <0.0001 | 0.0003 | 0.0023 |
| 10^-7^ M | 28 ℃ vs. 34 ℃ | ** | ns | * | ns | 0.0023 | 0.1402 | 0.0204 | 0.2120 |
|  | 28 ℃ vs. 40 ℃ | * | **** | **** | *** | 0.0259 | <0.0001 | <0.0001 | 0.0002 |
|  | 34 ℃ vs. 40 ℃ | *** | *** | **** | *** | 0.0008 | 0.0001 | <0.0001 | 0.0003 |
| 10^-6^ M | 28 ℃ vs. 34 ℃ | ** | * | ns | ns | 0.0053 | 0.0174 | 0.8114 | 0.0838 |
|  | 28 ℃ vs. 40 ℃ | ** | **** | *** | *** | 0.0036 | <0.0001 | 0.0004 | 0.0007 |
|  | 34 ℃ vs. 40 ℃ | *** | **** | *** | ** | 0.0006 | <0.0001 | 0.0005 | 0.0012 |
| 10^-5^ M | 28 ℃ vs. 34 ℃ | * | ** | * | ** | 0.0131 | 0.0049 | 0.0157 | 0.0012 |
|  | 28 ℃ vs. 40 ℃ | ns | **** | **** | **** | 0.3205 | <0.0001 | <0.0001 | <0.0001 |
|  | 34 ℃ vs. 40 ℃ | ** | *** | **** | *** | 0.0068 | 0.0002 | <0.0001 | 0.0004 |
| 10^-4^ M | 28 ℃ vs. 34 ℃ | * | * | ns | * | 0.0174 | 0.0174 | 0.0560 | 0.0164 |
|  | 28 ℃ vs. 40 ℃ | * | *** | ** | ** | 0.0121 | 0.0007 | 0.0012 | 0.0024 |
|  | 34 ℃ vs. 40 ℃ | ** | ** | ** | * | 0.0020 | 0.0021 | 0.0027 | 0.0194 |
| 10^-3^ M | 28 ℃ vs. 34 ℃ | ** | *** | *** | ** | 0.0085 | 0.0003 | 0.0002 | 0.0074 |
|  | 28 ℃ vs. 40 ℃ | ** | **** | *** | ** | 0.0033 | <0.0001 | 0.0003 | 0.0041 |
|  | 34 ℃ vs. 40 ℃ | *** | *** | ns | ns | 0.0007 | 0.0004 | 0.1652 | 0.2767 |

*indicates *p*<0.05; **indicates *p*<0.01; ***indicates *p*<0.001; ****indicates *p*<0.0001; ns indicates not significant.

**Table S28.** Post-hoc comparisons of the putrescine concentration effect on φ_EO_ within each temperature.

| Dunnett's multiple comparisons test | | Summary | | | | Adjusted *p* value | | | |
| --- | --- | --- | --- | --- | --- | --- | --- | --- | --- |
|  |  | Day 2 | Day 4 | Day 6 | Day 8 | Day 2 | Day 4 | Day 6 | Day 8 |
| 28 ℃ | Control vs. 10^-7^ M | ns | ns | ns | ns | 0.7292 | 0.4257 | 0.9431 | 0.0935 |
|  | Control vs. 10^-6^ M | ns | ns | ns | ** | >0.9999 | 0.7466 | 0.1250 | 0.0024 |
|  | Control vs. 10^-5^ M | ns | ns | ns | ** | 0.6587 | 0.0673 | 0.9952 | 0.0032 |
|  | Control vs. 10^-4^ M | ns | ns | ns | * | 0.9576 | 0.0835 | 0.8873 | 0.0125 |
|  | Control vs. 10^-3^ M | * | ns | ** | ns | 0.0288 | 0.9567 | 0.0047 | 0.7055 |
|  | 10^-7^ M vs. 10^-6^ M | ns | ns | ns | * | 0.6666 | 0.9809 | 0.3253 | 0.0477 |
|  | 10^-7^ M vs. 10^-5^ M | ns | ns | ns | ns | >0.9999 | 0.5838 | 0.7566 | 0.0725 |
|  | 10^-7^ M vs. 10^-4^ M | ns | ns | ns | ns | 0.3551 | 0.6849 | 0.4767 | 0.4408 |
|  | 10^-7^ M vs. 10^-3^ M | ** | ns | ** | * | 0.0079 | 0.1787 | 0.0098 | 0.0208 |
|  | 10^-6^ M vs. 10^-5^ M | ns | ns | ns | ns | 0.5958 | 0.3047 | 0.0735 | 0.9982 |
|  | 10^-6^ M vs. 10^-4^ M | ns | ns | * | ns | 0.9785 | 0.3758 | 0.0403 | 0.4173 |
|  | 10^-6^ M vs. 10^-3^ M | * | ns | ns | *** | 0.0326 | 0.3668 | 0.0952 | 0.0009 |
|  | 10^-5^ M vs. 10^-4^ M | ns | ns | ns | ns | 0.3065 | >0.9999 | 0.9903 | 0.6009 |
|  | 10^-5^ M vs. 10^-3^M | ** | * | ** | ** | 0.0070 | 0.0291 | 0.0033 | 0.0012 |
|  | 10^-4^ M vs. 10^-3^ M | ns | * | ** | ** | 0.0663 | 0.0355 | 0.0022 | 0.0037 |
| 34 ℃ | Control vs. 10^-7^ M | * | ** | *** | ** | 0.0485 | 0.0064 | 0.0003 | 0.0026 |
|  | Control vs. 10^-6^ M | * | ns | ns | ** | 0.0156 | 0.6914 | >0.9999 | 0.0027 |
|  | Control vs. 10^-5^ M | ns | ns | ns | ns | 0.1612 | 0.2299 | 0.8862 | 0.1602 |
|  | Control vs. 10^-4^ M | ns | **** | ** | ns | 0.0629 | <0.0001 | 0.0040 | 0.9992 |
|  | Control vs. 10^-3^ M | ns | **** | **** | * | 0.8974 | <0.0001 | <0.0001 | 0.0439 |
|  | 10^-7^ M vs. 10^-6^ M | ns | * | *** | ns | 0.8499 | 0.0243 | 0.0004 | >0.9999 |
|  | 10^-7^ M vs. 10^-5^ M | ns | *** | *** | * | 0.8679 | 0.0010 | 0.0002 | 0.0325 |
|  | 10^-7^ M vs. 10^-4^ M | ns | **** | **** | ** | 0.9998 | <0.0001 | <0.0001 | 0.0033 |
|  | 10^-7^ M vs. 10^-3^ M | * | **** | **** | *** | 0.0175 | <0.0001 | <0.0001 | 0.0002 |
|  | 10^-6^ M vs. 10^-5^ M | ns | * | ns | * | 0.3419 | 0.0455 | 0.8181 | 0.0336 |
|  | 10^-6^ M vs. 10^-4^ M | ns | **** | ** | ** | 0.7368 | <0.0001 | 0.0036 | 0.0034 |
|  | 10^-6^ M vs. 10^-3^ M | ** | **** | **** | *** | 0.0064 | <0.0001 | <0.0001 | 0.0002 |
|  | 10^-5^ M vs. 10^-4^ M | ns | *** | ** | ns | 0.9460 | 0.0002 | 0.0096 | 0.2317 |
|  | 10^-5^ M vs. 10^-3^M | ns | **** | **** | ** | 0.0527 | <0.0001 | <0.0001 | 0.0032 |
|  | 10^-4^ M vs. 10^-3^ M | * | **** | **** | * | 0.0222 | <0.0001 | <0.0001 | 0.0313 |
| 40 ℃ | Control vs. 10^-7^ M | ns | ns | ns | ns | 0.1554 | 0.9998 | 0.9965 | 0.9965 |
|  | Control vs. 10^-6^ M | ns | ** | ns | ns | 0.8451 | 0.0041 | 0.6390 | 0.6390 |
|  | Control vs. 10^-5^ M | ns | ns | ns | ns | 0.6321 | 0.1199 | 0.9922 | >0.9999 |
|  | Control vs. 10^-4^ M | ns | ** | ns | ns | 0.3571 | 0.0058 | 0.8191 | 0.9649 |
|  | Control vs. 10^-3^ M | **** | **** | ns | ns | <0.0001 | <0.0001 | 0.7500 | 0.9961 |
|  | 10^-7^ M vs. 10^-6^ M | ns | ** | ns | ns | 0.5271 | 0.0049 | 0.8547 | 0.8547 |
|  | 10^-7^ M vs. 10^-5^ M | ns | ns | ns | ns | 0.7481 | 0.1568 | 0.9084 | 0.9980 |
|  | 10^-7^ M vs. 10^-4^ M | * | ** | ns | ns | 0.0157 | 0.0048 | 0.5968 | 0.8193 |
|  | 10^-7^ M vs. 10^-3^ M | **** | **** | ns | ns | <0.0001 | <0.0001 | 0.9308 | >0.9999 |
|  | 10^-6^ M vs. 10^-5^ M | ns | ns | ns | ns | 0.9969 | 0.0807 | 0.3889 | 0.6637 |
|  | 10^-6^ M vs. 10^-4^ M | ns | *** | ns | ns | 0.1001 | 0.0001 | 0.1866 | 0.3060 |
|  | 10^-6^ M vs. 10^-3^ M | **** | **** | ns | ns | <0.0001 | <0.0001 | 0.9999 | 0.8595 |
|  | 10^-5^ M vs. 10^-4^ M | ns | *** | ns | ns | 0.0619 | 0.0006 | 0.9785 | 0.9552 |
|  | 10^-5^ M vs. 10^-3^M | **** | **** | ns | ns | <0.0001 | <0.0001 | 0.4834 | 0.9977 |
|  | 10^-4^ M vs. 10^-3^ M | **** | *** | ns | ns | <0.0001 | 0.0004 | 0.2381 | 0.8139 |

*indicates *p*<0.05; **indicates *p*<0.01; ***indicates *p*<0.001; ****indicates *p*<0.0001; ns indicates not significant.

**Table S29.** Two-way ANOVA results for the effects of temperature and putrescine concentration on δ_RO_.

| Factors | Day 2 | | Day 4 | | Day 6 | | Day 8 | |
| --- | --- | --- | --- | --- | --- | --- | --- | --- |
|  | F | *p* | F | *p* | F | *p* | F | *p* |
| Temperature | 28.191 | <0.001 | 64.258 | <0.001 | 2059.698 | <0.001 | 188.462 | <0.001 |
| Putrescine | 2.451 | 0.073 | 0.546 | 0.092 | 87.372 | <0.001 | 2.060 | <0.001 |
| Temperature* Putrescine | 2.196 | 0.070 | 0.246 | 0.092 | 36.837 | <0.001 | 1.285 | <0.001 |

**Table S30.** Post-hoc comparisons of the temperature effect on δ_RO_ within each putrescine concentration.

| Dunnett's multiple comparisons test | | Summary | | | | Adjusted *p* value | | | |
| --- | --- | --- | --- | --- | --- | --- | --- | --- | --- |
|  |  | Day 2 | Day 4 | Day 6 | Day 8 | Day 2 | Day 4 | Day 6 | Day 8 |
| 0 M | 28 ℃ vs. 34 ℃ | ns | * | ns | ns | 0.8402 | 0.0269 | 0.9874 | 0.9270 |
|  | 28 ℃ vs. 40 ℃ | ns | *** | ns | ns | 0.7081 | 0.0007 | 0.5924 | 0.4712 |
|  | 34 ℃ vs. 40 ℃ | ns | ** | ns | ns | 0.9636 | 0.0017 | 0.6689 | 0.6471 |
| 10^-7^ M | 28 ℃ vs. 34 ℃ | ** | ns | ns | ns | 0.0036 | 0.2120 | 0.9255 | 0.9256 |
|  | 28 ℃ vs. 40 ℃ | ns | *** | * | * | 0.5105 | 0.0005 | 0.0432 | 0.0195 |
|  | 34 ℃ vs. 40 ℃ | ** | *** | ns | * | 0.0051 | 0.0006 | 0.0546 | 0.0234 |
| 10^-6^ M | 28 ℃ vs. 34 ℃ | ** | * | ns | ns | 0.0030 | 0.0154 | 0.9961 | 0.9784 |
|  | 28 ℃ vs. 40 ℃ | ns | **** | ns | ns | 0.0581 | <0.0001 | 0.7717 | 0.3059 |
|  | 34 ℃ vs. 40 ℃ | ** | **** | ns | ns | 0.0013 | <0.0001 | 0.8141 | 0.3665 |
| 10^-5^ M | 28 ℃ vs. 34 ℃ | ** | * | ns | ns | 0.0076 | 0.0131 | 0.0697 | 0.9012 |
|  | 28 ℃ vs. 40 ℃ | ns | *** | *** | ns | 0.8334 | 0.0002 | 0.0006 | 0.1486 |
|  | 34 ℃ vs. 40 ℃ | ** | *** | *** | ns | 0.0094 | 0.0006 | 0.0010 | 0.2156 |
| 10^-4^ M | 28 ℃ vs. 34 ℃ | ** | * | ns | ns | 0.0030 | 0.0456 | 0.9735 | 0.9344 |
|  | 28 ℃ vs. 40 ℃ | ** | *** | ns | ns | 0.0074 | 0.0007 | 0.4815 | 0.2012 |
|  | 34 ℃ vs. 40 ℃ | *** | ** | ns | ns | 0.0006 | 0.0013 | 0.5826 | 0.2748 |
| 10^-3^ M | 28 ℃ vs. 34 ℃ | ** | ns | ns | ns | 0.0088 | 0.7903 | 0.2798 | 0.9505 |
|  | 28 ℃ vs. 40 ℃ | ** | ns | ns | ns | 0.0081 | 0.2673 | 0.1281 | 0.0728 |
|  | 34 ℃ vs. 40 ℃ | ns | ns | ns | ns | 0.9784 | 0.4925 | 0.6653 | 0.0594 |

*indicates *p*<0.05; **indicates *p*<0.01; ***indicates *p*<0.001; ****indicates *p*<0.0001; ns indicates not significant.

**Table S31.** Post-hoc comparisons of the putrescine concentration effect on δ_RO_ within each temperature.

| Dunnett's multiple comparisons test | | Summary | | | | Adjusted *p* value | | | |
| --- | --- | --- | --- | --- | --- | --- | --- | --- | --- |
|  |  | Day 2 | Day 4 | Day 6 | Day 8 | Day 2 | Day 4 | Day 6 | Day 8 |
| 28 ℃ | Control vs. 10^-7^ M | ns | ns | ** | ns | 0.7082 | 0.3047 | 0.0053 | 0.0670 |
|  | Control vs. 10^-6^ M | ns | ns | ns | ** | 0.9727 | 0.4501 | 0.7932 | 0.0068 |
|  | Control vs. 10^-5^ M | ns | ns | ** | * | 0.6441 | 0.7474 | 0.0046 | 0.0181 |
|  | Control vs. 10^-4^ M | ns | ns | ** | * | 0.9901 | 0.5589 | 0.0019 | 0.0247 |
|  | Control vs. 10^-3^ M | ns | ns | ns | ** | 0.9407 | 0.1965 | 0.3621 | 0.0046 |
|  | 10^-7^ M vs. 10^-6^ M | ns | ns | * | ns | 0.9715 | 0.9985 | 0.0159 | 0.2907 |
|  | 10^-7^ M vs. 10^-5^ M | ns | ns | ns | ns | >0.9999 | 0.0674 | >0.9999 | 0.7854 |
|  | 10^-7^ M vs. 10^-4^ M | ns | * | ns | ns | 0.9397 | 0.0453 | 0.7563 | 0.9140 |
|  | 10^-7^ M vs. 10^-3^ M | ns | ns | * | ns | 0.9898 | 0.9980 | 0.0392 | 0.1726 |
|  | 10^-6^ M vs. 10^-5^ M | ns | ns | * | ns | 0.9463 | 0.1019 | 0.0136 | 0.8651 |
|  | 10^-6^ M vs. 10^-4^ M | ns | ns | ** | ns | >0.9999 | 0.0677 | 0.0050 | 0.7183 |
|  | 10^-6^ M vs. 10^-3^ M | ns | ns | ns | ns | >0.9999 | 0.9625 | 0.9299 | 0.9957 |
|  | 10^-5^ M vs. 10^-4^ M | ns | ns | ns | ns | 0.9021 | 0.9985 | 0.8373 | 0.9993 |
|  | 10^-5^ M vs. 10^-3^M | ns | * | * | ns | 0.9761 | 0.0441 | 0.0328 | 0.6407 |
|  | 10^-4^ M vs. 10^-3^ M | ns | * | * | ns | 0.9994 | 0.0301 | 0.0107 | 0.4838 |
| 34 ℃ | Control vs. 10^-7^ M | ns | ** | ** | ns | 0.6846 | 0.0020 | 0.0041 | 0.8197 |
|  | Control vs. 10^-6^ M | ns | ** | ns | ns | 0.9681 | 0.0072 | 0.8048 | 0.8301 |
|  | Control vs. 10^-5^ M | ns | * | ns | ns | 0.9922 | 0.0468 | 0.1701 | 0.9664 |
|  | Control vs. 10^-4^ M | ns | ns | ns | ns | 0.5024 | 0.5278 | 0.6117 | 0.9649 |
|  | Control vs. 10^-3^ M | ns | *** | **** | ns | 0.9984 | 0.0003 | <0.0001 | 0.4044 |
|  | 10^-7^ M vs. 10^-6^ M | ns | ns | * | ns | 0.9684 | 0.6059 | 0.0115 | >0.9999 |
|  | 10^-7^ M vs. 10^-5^ M | ns | ns | ns | ns | 0.4255 | 0.0747 | 0.0572 | 0.9963 |
|  | 10^-7^ M vs. 10^-4^ M | ns | ** | * | ns | 0.9987 | 0.0083 | 0.0164 | 0.9966 |
|  | 10^-7^ M vs. 10^-3^ M | ns | **** | **** | ns | 0.4950 | <0.0001 | <0.0001 | 0.9430 |
|  | 10^-6^ M vs. 10^-5^ M | ns | ns | ns | ns | 0.7875 | 0.4489 | 0.6127 | 0.9971 |
|  | 10^-6^ M vs. 10^-4^ M | ns | * | ns | ns | 0.8632 | 0.0392 | 0.9982 | 0.9973 |
|  | 10^-6^ M vs. 10^-3^ M | ns | **** | **** | ns | 0.8562 | <0.0001 | <0.0001 | 0.9369 |
|  | 10^-5^ M vs. 10^-4^ M | ns | ns | ns | ns | 0.2906 | 0.3368 | 0.8058 | >0.9999 |
|  | 10^-5^ M vs. 10^-3^M | ns | **** | **** | ns | >0.9999 | <0.0001 | <0.0001 | 0.7687 |
|  | 10^-4^ M vs. 10^-3^ M | ns | *** | **** | ns | 0.3431 | 0.0001 | <0.0001 | 0.7728 |
| 40 ℃ | Control vs. 10^-7^ M | **** | ns | ns | ns | <0.0001 | >0.9999 | 0.9997 | 0.9996 |
|  | Control vs. 10^-6^ M | **** | ns | ns | ns | <0.0001 | >0.9999 | >0.9999 | >0.9999 |
|  | Control vs. 10^-5^ M | **** | ns | ns | ns | <0.0001 | >0.9999 | >0.9999 | >0.9999 |
|  | Control vs. 10^-4^ M | **** | ns | ns | ns | <0.0001 | 0.9874 | >0.9999 | >0.9999 |
|  | Control vs. 10^-3^ M | ns | ns | ns | ns | 0.7157 | 0.9965 | >0.9999 | >0.9999 |
|  | 10^-7^ M vs. 10^-6^ M | ns | ns | ns | ns | >0.9999 | >0.9999 | 0.9993 | >0.9999 |
|  | 10^-7^ M vs. 10^-5^ M | ns | ns | ns | ns | 0.8963 | >0.9999 | >0.9999 | 0.9983 |
|  | 10^-7^ M vs. 10^-4^ M | ns | ns | ns | ns | 0.9989 | 0.9871 | 0.9959 | 0.9971 |
|  | 10^-7^ M vs. 10^-3^ M | *** | ns | ns | ns | 0.0001 | 0.9964 | 0.9981 | 0.9984 |
|  | 10^-6^ M vs. 10^-5^ M | ns | ns | ns | ns | 0.8226 | >0.9999 | >0.9999 | 0.9998 |
|  | 10^-6^ M vs. 10^-4^ M | ns | ns | ns | ns | 0.9923 | 0.9826 | >0.9999 | 0.9996 |
|  | 10^-6^ M vs. 10^-3^ M | *** | ns | ns | ns | 0.0001 | 0.9946 | >0.9999 | 0.9999 |
|  | 10^-5^ M vs. 10^-4^ M | ns | ns | ns | ns | 0.9794 | 0.9688 | 0.9997 | >0.9999 |
|  | 10^-5^ M vs. 10^-3^M | **** | ns | ns | ns | <0.0001 | 0.9880 | >0.9999 | >0.9999 |
|  | 10^-4^ M vs. 10^-3^ M | **** | ns | ns | ns | <0.0001 | >0.9999 | >0.9999 | >0.9999 |

*indicates *p*<0.05; **indicates *p*<0.01; ***indicates *p*<0.001; ****indicates *p*<0.0001; ns indicates not significant.

**Table S32.** Two-way ANOVA results for the effects of temperature and putrescine concentration on PI_ABS_.

| Factors | Day 2 | | Day 4 | | Day 6 | | Day 8 | |
| --- | --- | --- | --- | --- | --- | --- | --- | --- |
|  | F | *p* | F | *p* | F | *p* | F | *p* |
| Temperature | 33.011 | <0.001 | 272.159 | <0.001 | 757.736 | <0.001 | 410.495 | <0.001 |
| Putrescine | 7.427 | <0.001 | 10.263 | <0.001 | 42.869 | <0.001 | 13.313 | <0.001 |
| Temperature* Putrescine | 2.535 | <0.05 | 5.435 | <0.001 | 35.285 | <0.001 | 5.997 | <0.001 |

**Table S33.** Post-hoc comparisons of the temperature effect on PI_ABS_ within each putrescine concentration.

| Dunnett's multiple comparisons test | | Summary | | | | Adjusted *p* value | | | |
| --- | --- | --- | --- | --- | --- | --- | --- | --- | --- |
|  |  | Day 2 | Day 4 | Day 6 | Day 8 | Day 2 | Day 4 | Day 6 | Day 8 |
| 0 M | 28 ℃ vs. 34 ℃ | ns | ns | ** | **** | 0.2901 | 0.0731 | 0.0037 | <0.0001 |
|  | 28 ℃ vs. 40 ℃ | ns | ** | *** | **** | 0.2210 | 0.0075 | 0.0002 | <0.0001 |
|  | 34 ℃ vs. 40 ℃ | ns | * | ** | *** | 0.9502 | 0.0367 | 0.0012 | 0.0006 |
| 10^-7^ M | 28 ℃ vs. 34 ℃ | * | ns | * | * | 0.0358 | 0.6298 | 0.0208 | 0.0218 |
|  | 28 ℃ vs. 40 ℃ | * | ** | *** | ** | 0.0175 | 0.0029 | 0.0007 | 0.0015 |
|  | 34 ℃ vs. 40 ℃ | ns | ** | *** | ** | 0.4391 | 0.0037 | 0.0003 | 0.0060 |
| 10^-6^ M | 28 ℃ vs. 34 ℃ | ns | ** | ns | * | 0.0522 | 0.0059 | 0.1514 | 0.0114 |
|  | 28 ℃ vs. 40 ℃ | ns | **** | ** | ** | 0.0966 | <0.0001 | 0.0013 | 0.0018 |
|  | 34 ℃ vs. 40 ℃ | ns | *** | ** | * | 0.6747 | 0.0005 | 0.0022 | 0.0155 |
| 10^-5^ M | 28 ℃ vs. 34 ℃ | * | ** | * | * | 0.0403 | 0.0087 | 0.0101 | 0.0134 |
|  | 28 ℃ vs. 40 ℃ | * | ** | ** | ** | 0.0379 | 0.0018 | 0.0015 | 0.0061 |
|  | 34 ℃ vs. 40 ℃ | ns | * | * | ns | 0.9937 | 0.0207 | 0.0130 | 0.2267 |
| 10^-4^ M | 28 ℃ vs. 34 ℃ | ns | * | ** | * | 0.3250 | 0.0168 | 0.0052 | 0.0138 |
|  | 28 ℃ vs. 40 ℃ | * | ** | ** | ** | 0.0392 | 0.0052 | 0.0021 | 0.0058 |
|  | 34 ℃ vs. 40 ℃ | ns | ns | ns | ns | 0.1276 | 0.0987 | 0.0761 | 0.1900 |
| 10^-3^ M | 28 ℃ vs. 34 ℃ | ns | ** | *** | **** | 0.8406 | 0.0022 | 0.0005 | <0.0001 |
|  | 28 ℃ vs. 40 ℃ | ns | ** | *** | **** | 0.1648 | 0.0014 | 0.0005 | <0.0001 |
|  | 34 ℃ vs. 40 ℃ | ns | ns | ns | ns | 0.1043 | 0.2345 | 0.9893 | 0.4824 |

*indicates *p*<0.05; **indicates *p*<0.01; ***indicates *p*<0.001; ****indicates *p*<0.0001; ns indicates not significant.

**Table S34.** Post-hoc comparisons of the putrescine concentration effect on PI_ABS_ within each temperature.

| Dunnett's multiple comparisons test | | Summary | | | | Adjusted *p* value | | | |
| --- | --- | --- | --- | --- | --- | --- | --- | --- | --- |
|  |  | Day 2 | Day 4 | Day 6 | Day 8 | Day 2 | Day 4 | Day 6 | Day 8 |
| 28 ℃ | Control vs. 10^-7^ M | ns | ns | ns | ns | 0.7795 | 0.8069 | 0.9820 | 0.5910 |
|  | Control vs. 10^-6^ M | ns | ns | ns | ns | >0.9999 | 0.6929 | 0.1329 | 0.1495 |
|  | Control vs. 10^-5^ M | ns | ns | ns | * | 0.6385 | 0.0666 | 0.9453 | 0.0414 |
|  | Control vs. 10^-4^ M | ns | ns | ns | ns | 0.9978 | 0.0527 | 0.5651 | 0.5979 |
|  | Control vs. 10^-3^ M | ns | ns | ns | ns | 0.1986 | >0.9999 | 0.1004 | >0.9999 |
|  | 10^-7^ M vs. 10^-6^ M | ns | ns | ns | ns | 0.8269 | 0.9998 | 0.2736 | 0.7709 |
|  | 10^-7^ M vs. 10^-5^ M | ns | ns | ns | ns | 0.9996 | 0.2630 | 0.6769 | 0.2590 |
|  | 10^-7^ M vs. 10^-4^ M | ns | ns | ns | ns | 0.5749 | 0.2066 | 0.2957 | >0.9999 |
|  | 10^-7^ M vs. 10^-3^ M | * | ns | ns | ns | 0.0477 | 0.7935 | 0.2073 | 0.6118 |
|  | 10^-6^ M vs. 10^-5^ M | ns | ns | ns | ns | 0.6913 | 0.3381 | 0.0521 | 0.8338 |
|  | 10^-6^ M vs. 10^-4^ M | ns | ns | * | ns | 0.9938 | 0.2673 | 0.0216 | 0.7642 |
|  | 10^-6^ M vs. 10^-3^ M | ns | ns | ns | ns | 0.1770 | 0.6781 | 0.9998 | 0.1565 |
|  | 10^-5^ M vs. 10^-4^ M | ns | ns | ns | ns | 0.4424 | 0.9999 | 0.9417 | 0.2551 |
|  | 10^-5^ M vs. 10^-3^M | * | ns | * | * | 0.0357 | 0.0645 | 0.0401 | 0.0433 |
|  | 10^-4^ M vs. 10^-3^ M | ns | ns | * | ns | 0.3105 | 0.0511 | 0.0170 | 0.6188 |
| 34 ℃ | Control vs. 10^-7^ M | ns | *** | *** | ** | 0.3193 | 0.0003 | 0.0002 | 0.0074 |
|  | Control vs. 10^-6^ M | ns | ** | ns | * | 0.2809 | 0.0054 | >0.9999 | 0.0141 |
|  | Control vs. 10^-5^ M | ns | ns | ns | ns | 0.4964 | 0.0659 | 0.6134 | 0.3706 |
|  | Control vs. 10^-4^ M | * | ns | * | ns | 0.0377 | >0.9999 | 0.0134 | 0.7479 |
|  | Control vs. 10^-3^ M | ns | ** | *** | ns | 0.7618 | 0.0010 | 0.0003 | 0.5583 |
|  | 10^-7^ M vs. 10^-6^ M | ns | * | *** | ns | >0.9999 | 0.0337 | 0.0002 | 0.9701 |
|  | 10^-7^ M vs. 10^-5^ M | ns | ** | **** | ns | 0.9970 | 0.0033 | <0.0001 | 0.0584 |
|  | 10^-7^ M vs. 10^-4^ M | ns | *** | **** | * | 0.4551 | 0.0003 | <0.0001 | 0.0257 |
|  | 10^-7^ M vs. 10^-3^ M | ns | **** | **** | ** | 0.0729 | <0.0001 | <0.0001 | 0.0019 |
|  | 10^-6^ M vs. 10^-5^ M | ns | ns | ns | ns | 0.9910 | 0.2170 | 0.5647 | 0.1307 |
|  | 10^-6^ M vs. 10^-4^ M | ns | ** | * | ns | 0.5097 | 0.0061 | 0.0122 | 0.0546 |
|  | 10^-6^ M vs. 10^-3^ M | ns | **** | *** | ** | 0.0641 | <0.0001 | 0.0002 | 0.0033 |
|  | 10^-5^ M vs. 10^-4^ M | ns | ns | ns | ns | 0.2897 | 0.0776 | 0.0681 | 0.9583 |
|  | 10^-5^ M vs. 10^-3^M | ns | *** | *** | ns | 0.1179 | 0.0001 | 0.0006 | 0.0550 |
|  | 10^-4^ M vs. 10^-3^ M | * | *** | ** | ns | 0.0105 | 0.0010 | 0.0091 | 0.1318 |
| 40 ℃ | Control vs. 10^-7^ M | ns | ns | ns | ns | >0.9999 | 0.9988 | 0.9678 | 0.9966 |
|  | Control vs. 10^-6^ M | ns | ns | ns | ns | 0.4238 | 0.9989 | 0.9978 | 0.9682 |
|  | Control vs. 10^-5^ M | ns | ns | ns | ns | 0.8662 | 0.3615 | >0.9999 | >0.9999 |
|  | Control vs. 10^-4^ M | ns | ns | ns | ns | >0.9999 | 0.6326 | >0.9999 | 0.9992 |
|  | Control vs. 10^-3^ M | ns | ns | ns | ns | 0.8685 | 0.5318 | 0.9995 | 0.9998 |
|  | 10^-7^ M vs. 10^-6^ M | ns | ns | ns | ns | 0.4230 | 0.9738 | 0.9991 | 0.9995 |
|  | 10^-7^ M vs. 10^-5^ M | ns | ns | ns | ns | 0.8654 | 0.2452 | 0.9782 | 0.9992 |
|  | 10^-7^ M vs. 10^-4^ M | ns | ns | ns | ns | >0.9999 | 0.8112 | 0.9901 | >0.9999 |
|  | 10^-7^ M vs. 10^-3^ M | ns | ns | ns | ns | 0.8693 | 0.7138 | 0.9964 | >0.9999 |
|  | 10^-6^ M vs. 10^-5^ M | ns | ns | ns | ns | 0.9258 | 0.5115 | 0.9991 | 0.9846 |
|  | 10^-6^ M vs. 10^-4^ M | ns | ns | ns | ns | 0.3700 | 0.4624 | 0.9999 | 0.9975 |
|  | 10^-6^ M vs. 10^-3^ M | ns | ns | ns | ns | 0.1285 | 0.3777 | >0.9999 | 0.9940 |
|  | 10^-5^ M vs. 10^-4^ M | ns | ns | ns | ns | 0.8094 | 0.0628 | >0.9999 | >0.9999 |
|  | 10^-5^ M vs. 10^-3^M | ns | ns | ns | ns | 0.3572 | 0.0506 | 0.9999 | >0.9999 |
|  | 10^-4^ M vs. 10^-3^ M | ns | ns | ns | ns | 0.9155 | >0.9999 | >0.9999 | >0.9999 |

*indicates *p*<0.05; **indicates *p*<0.01; ***indicates *p*<0.001; ****indicates *p*<0.0001; ns indicates not significant.

**Table S35.** Two-way ANOVA results for the effects of temperature and putrescine concentration on ROS.

| Factors | Day 2 | | Day 4 | | Day 6 | | Day 8 | |
| --- | --- | --- | --- | --- | --- | --- | --- | --- |
|  | F | *p* | F | *p* | F | *p* | F | *p* |
| Temperature | 1293.415 | <0.001 | 3969.074 | <0.001 | 1318.210 | <0.001 | 695.140 | <0.001 |
| Putrescine | 174.141 | <0.001 | 72.885 | <0.001 | 7.780 | <0.001 | 43.848 | <0.001 |
| Temperature* Putrescine | 242.164 | <0.001 | 88.844 | <0.001 | 51.207 | <0.001 | 27.097 | <0.001 |

**Table S36.** Post-hoc comparisons of the temperature effect on ROS within each putrescine concentration.

| Dunnett's multiple comparisons test | | Summary | | | | Adjusted *p* value | | | |
| --- | --- | --- | --- | --- | --- | --- | --- | --- | --- |
|  |  | Day 2 | Day 4 | Day 6 | Day 8 | Day 2 | Day 4 | Day 6 | Day 8 |
| 0 M | 28 ℃ vs. 34 ℃ | **** | ns | **** | **** | <0.0001 | 0.6533 | <0.0001 | <0.0001 |
|  | 28 ℃ vs. 40 ℃ | **** | **** | **** | **** | <0.0001 | <0.0001 | <0.0001 | <0.0001 |
|  | 34 ℃ vs. 40 ℃ | **** | **** | * | **** | <0.0001 | <0.0001 | 0.0318 | <0.0001 |
| 10^-7^ M | 28 ℃ vs. 34 ℃ | **** | ns | **** | **** | <0.0001 | 0.1848 | <0.0001 | <0.0001 |
|  | 28 ℃ vs. 40 ℃ | **** | **** | **** | *** | <0.0001 | <0.0001 | <0.0001 | 0.0004 |
|  | 34 ℃ vs. 40 ℃ | ns | **** | *** | ns | 0.0514 | <0.0001 | 0.0010 | 0.0526 |
| 10^-6^ M | 28 ℃ vs. 34 ℃ | *** | ns | **** | **** | 0.0001 | 0.2135 | <0.0001 | <0.0001 |
|  | 28 ℃ vs. 40 ℃ | **** | **** | **** | **** | <0.0001 | <0.0001 | <0.0001 | <0.0001 |
|  | 34 ℃ vs. 40 ℃ | **** | **** | **** | ns | <0.0001 | <0.0001 | <0.0001 | 0.6876 |
| 10^-5^ M | 28 ℃ vs. 34 ℃ | * | *** | **** | **** | 0.0361 | 0.0004 | <0.0001 | <0.0001 |
|  | 28 ℃ vs. 40 ℃ | **** | **** | **** | **** | <0.0001 | <0.0001 | <0.0001 | <0.0001 |
|  | 34 ℃ vs. 40 ℃ | **** | **** | **** | **** | <0.0001 | <0.0001 | <0.0001 | <0.0001 |
| 10^-4^ M | 28 ℃ vs. 34 ℃ | **** | ns | **** | **** | <0.0001 | 0.9969 | <0.0001 | <0.0001 |
|  | 28 ℃ vs. 40 ℃ | **** | **** | **** | **** | <0.0001 | <0.0001 | <0.0001 | <0.0001 |
|  | 34 ℃ vs. 40 ℃ | ns | **** | **** | **** | 0.3149 | <0.0001 | <0.0001 | <0.0001 |
| 10^-3^ M | 28 ℃ vs. 34 ℃ | **** | ns | **** | **** | <0.0001 | 0.3188 | <0.0001 | <0.0001 |
|  | 28 ℃ vs. 40 ℃ | *** | **** | **** | **** | 0.0002 | <0.0001 | <0.0001 | <0.0001 |
|  | 34 ℃ vs. 40 ℃ | ** | **** | **** | ** | 0.0014 | <0.0001 | <0.0001 | 0.0014 |

*indicates *p*<0.05; **indicates *p*<0.01; ***indicates *p*<0.001; ****indicates *p*<0.0001; ns indicates not significant.

**Table S37.** Two-way ANOVA results for the effects of temperature and putrescine concentration on O_2_^-^.

| Factors | Day 2 | | Day 4 | | Day 6 | | Day 8 | |
| --- | --- | --- | --- | --- | --- | --- | --- | --- |
|  | F | *p* | F | *p* | F | *p* | F | *p* |
| Temperature | 393.504 | <0.001 | 347.889 | <0.001 | 801.149 | <0.001 | 200.118 | <0.001 |
| Putrescine | 13.170 | <0.001 | 108.868 | <0.001 | 82.401 | <0.001 | 23.214 | <0.001 |
| Temperature* Putrescine | 40.432 | <0.001 | 68.616 | <0.001 | 131.520 | <0.001 | 16.495 | <0.001 |

**Table S38.** Post-hoc comparisons of the temperature effect on O_2_^-^ within each putrescine concentration.

| Dunnett's multiple comparisons test | | Summary | | | | Adjusted *p* value | | | |
| --- | --- | --- | --- | --- | --- | --- | --- | --- | --- |
|  |  | Day 2 | Day 4 | Day 6 | Day 8 | Day 2 | Day 4 | Day 6 | Day 8 |
| 0 M | 28 ℃ vs. 34 ℃ | ** | ns | **** | **** | 0.0045 | 0.8148 | <0.0001 | <0.0001 |
|  | 28 ℃ vs. 40 ℃ | ns | **** | **** | **** | 0.5968 | <0.0001 | <0.0001 | <0.0001 |
|  | 34 ℃ vs. 40 ℃ | ** | **** | *** | **** | 0.0018 | <0.0001 | 0.0003 | <0.0001 |
| 10^-7^ M | 28 ℃ vs. 34 ℃ | **** | ns | ** | **** | <0.0001 | 0.7660 | 0.0014 | <0.0001 |
|  | 28 ℃ vs. 40 ℃ | **** | **** | **** | **** | <0.0001 | <0.0001 | <0.0001 | <0.0001 |
|  | 34 ℃ vs. 40 ℃ | *** | **** | ** | **** | 0.0001 | <0.0001 | 0.0016 | <0.0001 |
| 10^-6^ M | 28 ℃ vs. 34 ℃ | **** | * | **** | **** | <0.0001 | 0.0174 | <0.0001 | <0.0001 |
|  | 28 ℃ vs. 40 ℃ | *** | **** | ns | *** | 0.0006 | <0.0001 | 0.4448 | 0.0004 |
|  | 34 ℃ vs. 40 ℃ | * | **** | **** | **** | 0.0264 | <0.0001 | <0.0001 | <0.0001 |
| 10^-5^ M | 28 ℃ vs. 34 ℃ | ns | ** | **** | *** | 0.0832 | 0.0038 | <0.0001 | 0.0007 |
|  | 28 ℃ vs. 40 ℃ | ns | * | **** | ns | 0.0589 | 0.0264 | <0.0001 | 0.3062 |
|  | 34 ℃ vs. 40 ℃ | ns | ns | **** | *** | 0.9593 | 0.2301 | <0.0001 | 0.0002 |
| 10^-4^ M | 28 ℃ vs. 34 ℃ | **** | **** | **** | ns | <0.0001 | <0.0001 | <0.0001 | 0.1681 |
|  | 28 ℃ vs. 40 ℃ | ns | * | ns | ns | 0.1812 | 0.0135 | 0.0947 | 0.0520 |
|  | 34 ℃ vs. 40 ℃ | **** | **** | **** | ** | <0.0001 | <0.0001 | <0.0001 | 0.0051 |
| 10^-3^ M | 28 ℃ vs. 34 ℃ | **** | ns | ns | ns | <0.0001 | >0.9999 | 0.2966 | 0.8513 |
|  | 28 ℃ vs. 40 ℃ | *** | ns | * | *** | 0.0005 | 0.5813 | 0.0461 | 0.0001 |
|  | 34 ℃ vs. 40 ℃ | **** | ns | ** | **** | <0.0001 | 0.5780 | 0.0073 | <0.0001 |

*indicates *p*<0.05; **indicates *p*<0.01; ***indicates *p*<0.001; ****indicates *p*<0.0001; ns indicates not significant.

**Table S39.** Two-way ANOVA results for the effects of temperature and putrescine concentration on H_2_O_2_.

| Factors | Day 2 | | Day 4 | | Day 6 | | Day 8 | |
| --- | --- | --- | --- | --- | --- | --- | --- | --- |
|  | F | *p* | F | *p* | F | *p* | F | *p* |
| Temperature | 15.302 | <0.001 | 43.839 | <0.001 | 9077.974 | <0.001 | 1020.322 | <0.001 |
| Putrescine | 9.739 | <0.001 | 5.845 | <0.001 | 881.027 | <0.001 | 104.190 | <0.001 |
| Temperature* Putrescine | 6.029 | <0.001 | 7.144 | <0.001 | 654.890 | <0.001 | 93.931 | <0.001 |

**Table S40.** Post-hoc comparisons of the temperature effect on H_2_O_2_ within each putrescine concentration.

| Dunnett's multiple comparisons test | | Summary | | | | Adjusted *p* value | | | |
| --- | --- | --- | --- | --- | --- | --- | --- | --- | --- |
|  |  | Day 2 | Day 4 | Day 6 | Day 8 | Day 2 | Day 4 | Day 6 | Day 8 |
| 0 M | 28 ℃ vs. 34 ℃ | * | **** | **** | **** | 0.0304 | <0.0001 | <0.0001 | <0.0001 |
|  | 28 ℃ vs. 40 ℃ | **** | ** | * | ns | <0.0001 | 0.0016 | 0.0222 | 0.9996 |
|  | 34 ℃ vs. 40 ℃ | **** | **** | **** | **** | <0.0001 | <0.0001 | <0.0001 | <0.0001 |
| 10^-7^ M | 28 ℃ vs. 34 ℃ | ns | ** | **** | **** | 0.0667 | 0.0088 | <0.0001 | <0.0001 |
|  | 28 ℃ vs. 40 ℃ | ns | * | ns | ns | 0.9900 | 0.0158 | 0.1396 | 0.0552 |
|  | 34 ℃ vs. 40 ℃ | ns | *** | **** | **** | 0.0791 | 0.0003 | <0.0001 | <0.0001 |
| 10^-6^ M | 28 ℃ vs. 34 ℃ | **** | * | **** | **** | <0.0001 | 0.0182 | <0.0001 | <0.0001 |
|  | 28 ℃ vs. 40 ℃ | *** | ns | ns | *** | 0.0003 | 0.9775 | 0.3028 | 0.0009 |
|  | 34 ℃ vs. 40 ℃ | **** | * | **** | *** | <0.0001 | 0.0145 | <0.0001 | 0.0002 |
| 10^-5^ M | 28 ℃ vs. 34 ℃ | ns | **** | **** | **** | 0.1464 | <0.0001 | <0.0001 | <0.0001 |
|  | 28 ℃ vs. 40 ℃ | ns | * | **** | ** | 0.0631 | 0.0133 | <0.0001 | 0.0100 |
|  | 34 ℃ vs. 40 ℃ | ns | **** | **** | **** | 0.7923 | <0.0001 | <0.0001 | <0.0001 |
| 10^-4^ M | 28 ℃ vs. 34 ℃ | **** | **** | **** | **** | <0.0001 | <0.0001 | <0.0001 | <0.0001 |
|  | 28 ℃ vs. 40 ℃ | ** | ns | *** | * | 0.0041 | 0.1448 | 0.0005 | 0.0486 |
|  | 34 ℃ vs. 40 ℃ | **** | **** | **** | **** | <0.0001 | <0.0001 | <0.0001 | <0.0001 |
| 10^-3^ M | 28 ℃ vs. 34 ℃ | ns | **** | ** | **** | 0.8418 | <0.0001 | 0.0034 | <0.0001 |
|  | 28 ℃ vs. 40 ℃ | **** | **** | ns | ns | <0.0001 | <0.0001 | 0.0913 | 0.0726 |
|  | 34 ℃ vs. 40 ℃ | **** | **** | ns | *** | <0.0001 | <0.0001 | 0.0545 | 0.0002 |

*indicates *p*<0.05; **indicates *p*<0.01; ***indicates *p*<0.001; ****indicates *p*<0.0001; ns indicates not significant.

**Table S41.** Two-way ANOVA results for the effects of temperature and putrescine concentration on SOD.

| Factors | Day 2 | | Day 4 | | Day 6 | | Day 8 | |
| --- | --- | --- | --- | --- | --- | --- | --- | --- |
|  | F | *p* | F | *p* | F | *p* | F | *p* |
| Temperature | 139.525 | <0.001 | 78.314 | <0.001 | 216.624 | <0.001 | 333.126 | <0.001 |
| Putrescine | 62.759 | <0.001 | 81.571 | <0.001 | 38.825 | <0.001 | 50.982 | <0.001 |
| Temperature* Putrescine | 24.818 | <0.001 | 74.920 | <0.001 | 14.881 | <0.001 | 18.648 | <0.001 |

**Table S42.** Post-hoc comparisons of the temperature effect on SOD within each putrescine concentration.

| Dunnett's multiple comparisons test | | Summary | | | | Adjusted *p* value | | | |
| --- | --- | --- | --- | --- | --- | --- | --- | --- | --- |
|  |  | Day 2 | Day 4 | Day 6 | Day 8 | Day 2 | Day 4 | Day 6 | Day 8 |
| 0 M | 28 ℃ vs. 34 ℃ | **** | ns | *** | *** | <0.0001 | 0.9382 | 0.0001 | 0.0009 |
|  | 28 ℃ vs. 40 ℃ | **** | * | **** | ** | <0.0001 | 0.0254 | <0.0001 | 0.0016 |
|  | 34 ℃ vs. 40 ℃ | ns | * | ** | **** | 0.7035 | 0.0172 | 0.0012 | <0.0001 |
| 10^-7^ M | 28 ℃ vs. 34 ℃ | * | **** | *** | ns | 0.0161 | <0.0001 | 0.0007 | 0.1593 |
|  | 28 ℃ vs. 40 ℃ | ns | ns | *** | * | 0.0896 | 0.0960 | 0.0001 | 0.0102 |
|  | 34 ℃ vs. 40 ℃ | ** | **** | ns | ** | 0.0014 | <0.0001 | 0.1024 | 0.0014 |
| 10^-6^ M | 28 ℃ vs. 34 ℃ | *** | *** | **** | ns | 0.0003 | 0.0004 | <0.0001 | 0.3183 |
|  | 28 ℃ vs. 40 ℃ | *** | *** | **** | *** | 0.0007 | 0.0010 | <0.0001 | 0.0007 |
|  | 34 ℃ vs. 40 ℃ | **** | ns | ns | *** | <0.0001 | 0.4913 | 0.5518 | 0.0002 |
| 10^-5^ M | 28 ℃ vs. 34 ℃ | ns | ** | ns | ns | 0.7395 | 0.0075 | 0.7657 | 0.1462 |
|  | 28 ℃ vs. 40 ℃ | *** | ** | ** | *** | 0.0010 | 0.0040 | 0.0016 | 0.0002 |
|  | 34 ℃ vs. 40 ℃ | ** | ns | *** | ** | 0.0018 | 0.7958 | 0.0009 | 0.0011 |
| 10^-4^ M | 28 ℃ vs. 34 ℃ | * | ** | ns | *** | 0.0190 | 0.0017 | 0.9227 | 0.0004 |
|  | 28 ℃ vs. 40 ℃ | **** | *** | *** | **** | <0.0001 | 0.0003 | 0.0009 | <0.0001 |
|  | 34 ℃ vs. 40 ℃ | *** | ns | *** | **** | 0.0006 | 0.0909 | 0.0007 | <0.0001 |
| 10^-3^ M | 28 ℃ vs. 34 ℃ | * | ns | *** | ns | 0.0383 | 0.0831 | 0.0001 | 0.1507 |
|  | 28 ℃ vs. 40 ℃ | ns | ns | *** | **** | 0.0800 | 0.8885 | 0.0002 | <0.0001 |
|  | 34 ℃ vs. 40 ℃ | ** | ns | ns | **** | 0.0024 | 0.1509 | 0.9136 | <0.0001 |

*indicates *p*<0.05; **indicates *p*<0.01; ***indicates *p*<0.001; ****indicates *p*<0.0001; ns indicates not significant.

**Table S43.** Two-way ANOVA results for the effects of temperature and putrescine concentration on CAT.

| Factors | Day 2 | | Day 4 | | Day 6 | | Day 8 | |
| --- | --- | --- | --- | --- | --- | --- | --- | --- |
|  | F | *p* | F | *p* | F | *p* | F | *p* |
| Temperature | 2.929 | 0.066 | 24.502 | <0.001 | 6.715 | <0.05 | 8.189 | <0.05 |
| Putrescine | 1.832 | 0.092 | 2.475 | 0.050 | 1.488 | 0.092 | 1.703 | 0.092 |
| Temperature* Putrescine | 1.039 | .092 | 5.328 | <0.001 | 1.481 | 0.092 | 2.014 | 0.061 |

**Table S44.** Post-hoc comparisons of the temperature effect on CAT within each putrescine concentration.

| Dunnett's multiple comparisons test | | Summary | | | | Adjusted *p* value | | | |
| --- | --- | --- | --- | --- | --- | --- | --- | --- | --- |
|  |  | Day 2 | Day 4 | Day 6 | Day 8 | Day 2 | Day 4 | Day 6 | Day 8 |
| 0 M | 28 ℃ vs. 34 ℃ | ns | ns | ns | ns | 0.9827 | 0.0589 | 0.6267 | 0.1506 |
|  | 28 ℃ vs. 40 ℃ | ns | *** | ns | ns | 0.2579 | 0.0004 | 0.0734 | 0.9461 |
|  | 34 ℃ vs. 40 ℃ | ns | ** | ns | ns | 0.3200 | 0.0039 | 0.2472 | 0.2253 |
| 10^-7^ M | 28 ℃ vs. 34 ℃ | ns | * | ns | ns | 0.3834 | 0.0314 | 0.2348 | 0.0938 |
|  | 28 ℃ vs. 40 ℃ | ns | ns | ns | ns | 0.8939 | 0.0589 | 0.0623 | 0.3287 |
|  | 34 ℃ vs. 40 ℃ | ns | ns | ns | ns | 0.6154 | 0.8663 | 0.5773 | 0.6036 |
| 10^-6^ M | 28 ℃ vs. 34 ℃ | ns | * | ns | ns | 0.2647 | 0.0152 | 0.7958 | 0.1872 |
|  | 28 ℃ vs. 40 ℃ | ns | ns | ns | ns | 0.1495 | 0.7652 | 0.2195 | 0.3634 |
|  | 34 ℃ vs. 40 ℃ | ns | * | ns | ns | 0.8942 | 0.0345 | 0.4745 | 0.8536 |
| 10^-5^ M | 28 ℃ vs. 34 ℃ | ns | ns | ns | ns | 0.7002 | 0.8228 | 0.5289 | 0.1497 |
|  | 28 ℃ vs. 40 ℃ | ns | ns | ns | ns | 0.4000 | 0.1567 | 0.7382 | 0.9941 |
|  | 34 ℃ vs. 40 ℃ | ns | ns | ns | ns | 0.8415 | 0.3317 | 0.9265 | 0.1311 |
| 10^-4^ M | 28 ℃ vs. 34 ℃ | ns | ns | ** | ns | 0.2578 | 0.9244 | 0.0085 | 0.1461 |
|  | 28 ℃ vs. 40 ℃ | ns | ns | ns | ns | 0.9888 | 0.6748 | 0.4784 | 0.9967 |
|  | 34 ℃ vs. 40 ℃ | ns | ns | * | ns | 0.3067 | 0.8769 | 0.0334 | 0.1612 |
| 10^-3^ M | 28 ℃ vs. 34 ℃ | ns | ns | ns | ns | 0.2484 | 0.9974 | 0.9758 | 0.7198 |
|  | 28 ℃ vs. 40 ℃ | ns | ** | ns | ns | 0.8614 | 0.0078 | 0.4189 | 0.4676 |
|  | 34 ℃ vs. 40 ℃ | ns | ** | ns | ns | 0.1276 | 0.0083 | 0.5244 | 0.8918 |

*indicates *p*<0.05; **indicates *p*<0.01; ***indicates *p*<0.001; ****indicates *p*<0.0001; ns indicates not significant.

**Table S45.** Two-way ANOVA results for the effects of temperature and putrescine concentration on POD.

| Factors | Day 2 | | Day 4 | | Day 6 | | Day 8 | |
| --- | --- | --- | --- | --- | --- | --- | --- | --- |
|  | F | *p* | F | *p* | F | *p* | F | *p* |
| Temperature | 25.877 | <0.001 | 11.619 | <0.001 | 40.122 | <0.001 | 39.302 | <0.001 |
| Putrescine | 11.581 | <0.001 | 2.800 | <0.05 | 3.254 | <0.05 | 7.723 | <0.001 |
| Temperature* Putrescine | 4.486 | <0.001 | 1.950 | 0.069 | 4.095 | <0.001 | 3.764 | <0.05 |

**Table S46.** Post-hoc comparisons of the temperature effect on POD within each putrescine concentration.

| Dunnett's multiple comparisons test | | Summary | | | | Adjusted *p* value | | | |
| --- | --- | --- | --- | --- | --- | --- | --- | --- | --- |
|  |  | Day 2 | Day 4 | Day 6 | Day 8 | Day 2 | Day 4 | Day 6 | Day 8 |
| 0 M | 28 ℃ vs. 34 ℃ | * | **** | **** | ns | 0.0274 | <0.0001 | <0.0001 | 0.3462 |
|  | 28 ℃ vs. 40 ℃ | ns | **** | **** | ns | 0.1232 | <0.0001 | <0.0001 | 0.2200 |
|  | 34 ℃ vs. 40 ℃ | ns | **** | ** | ns | 0.4876 | <0.0001 | 0.0015 | 0.9272 |
| 10^-7^ M | 28 ℃ vs. 34 ℃ | ns | ** | * | *** | 0.4993 | 0.0031 | 0.0342 | 0.0002 |
|  | 28 ℃ vs. 40 ℃ | ns | ns | ns | ** | 0.1448 | 0.2716 | 0.6240 | 0.0089 |
|  | 34 ℃ vs. 40 ℃ | ns | * | ns | ** | 0.5848 | 0.0176 | 0.1132 | 0.0044 |
| 10^-6^ M | 28 ℃ vs. 34 ℃ | *** | ** | *** | **** | 0.0003 | 0.0014 | 0.0006 | <0.0001 |
|  | 28 ℃ vs. 40 ℃ | *** | ** | *** | *** | 0.0008 | 0.0063 | 0.0003 | 0.0006 |
|  | 34 ℃ vs. 40 ℃ | ns | ns | ns | ** | 0.3195 | 0.2965 | 0.4933 | 0.0061 |
| 10^-5^ M | 28 ℃ vs. 34 ℃ | ns | ns | * | **** | 0.1184 | 0.0612 | 0.0176 | <0.0001 |
|  | 28 ℃ vs. 40 ℃ | *** | * | ns | ns | 0.0006 | 0.0109 | 0.9969 | 0.3695 |
|  | 34 ℃ vs. 40 ℃ | ** | ns | * | **** | 0.0039 | 0.3590 | 0.0192 | <0.0001 |
| 10^-4^ M | 28 ℃ vs. 34 ℃ | *** | ns | * | *** | 0.0002 | 0.5595 | 0.0140 | 0.0001 |
|  | 28 ℃ vs. 40 ℃ | ** | ns | ns | * | 0.0041 | 0.1343 | 0.4536 | 0.0175 |
|  | 34 ℃ vs. 40 ℃ | * | ns | ns | ** | 0.0234 | 0.4926 | 0.0631 | 0.0018 |
| 10^-3^ M | 28 ℃ vs. 34 ℃ | * | ns | * | * | 0.0471 | 0.8253 | 0.0243 | 0.0282 |
|  | 28 ℃ vs. 40 ℃ | ns | ns | * | ns | 0.9847 | 0.0747 | 0.0136 | 0.1537 |
|  | 34 ℃ vs. 40 ℃ | ns | ns | ns | ns | 0.0580 | 0.1604 | 0.8669 | 0.4140 |

*indicates *p*<0.05; **indicates *p*<0.01; ***indicates *p*<0.001; ****indicates *p*<0.0001; ns indicates not significant.

**Table S47.** Two-way ANOVA results for the effects of temperature and putrescine concentration on GSH.

| Factors | Day 2 | | Day 4 | | Day 6 | | Day 8 | |
| --- | --- | --- | --- | --- | --- | --- | --- | --- |
|  | F | *p* | F | *p* | F | *p* | F | *p* |
| Temperature | 50.352 | <0.001 | 672.9 | <0.001 | 30.337 | <0.001 | 282.88 | <0.001 |
| Putrescine | 7.365 | <0.001 | 12.869 | <0.001 | 1.786 | 0.092 | 15.415 | <0.001 |
| Temperature* Putrescine | 5.747 | <0.001 | 17.045 | <0.001 | 1.461 | 0.092 | 15.997 | <0.001 |

**Table S48.** Post-hoc comparisons of the temperature effect on GSH within each putrescine concentration.

| Dunnett's multiple comparisons test | | Summary | | | | Adjusted *p* value | | | |
| --- | --- | --- | --- | --- | --- | --- | --- | --- | --- |
|  |  | Day 2 | Day 4 | Day 6 | Day 8 | Day 2 | Day 4 | Day 6 | Day 8 |
| 0 M | 28 ℃ vs. 34 ℃ | * | ns | * | *** | 0.0109 | 0.0565 | 0.0213 | 0.0009 |
|  | 28 ℃ vs. 40 ℃ | * | * | ns | ns | 0.0140 | 0.0137 | 0.0912 | 0.7716 |
|  | 34 ℃ vs. 40 ℃ | ns | ns | ns | *** | 0.8194 | 0.1393 | 0.1737 | 0.0010 |
| 10^-7^ M | 28 ℃ vs. 34 ℃ | ns | * | ns | ns | 0.1765 | 0.0480 | 0.0661 | 0.1036 |
|  | 28 ℃ vs. 40 ℃ | * | ** | ns | ns | 0.0127 | 0.0061 | 0.3732 | 0.7904 |
|  | 34 ℃ vs. 40 ℃ | * | * | ns | ns | 0.0419 | 0.0378 | 0.2263 | 0.1781 |
| 10^-6^ M | 28 ℃ vs. 34 ℃ | ns | ** | ns | ** | 0.6075 | 0.0049 | 0.2538 | 0.0017 |
|  | 28 ℃ vs. 40 ℃ | ns | **** | ns | ns | 0.0505 | <0.0001 | 0.9268 | 0.0517 |
|  | 34 ℃ vs. 40 ℃ | ns | *** | ns | ** | 0.1025 | 0.0005 | 0.3559 | 0.0048 |
| 10^-5^ M | 28 ℃ vs. 34 ℃ | ns | ns | ns | *** | 0.8376 | 0.2667 | 0.0921 | 0.0008 |
|  | 28 ℃ vs. 40 ℃ | * | ** | ns | ns | 0.0461 | 0.0011 | 0.9967 | 0.7567 |
|  | 34 ℃ vs. 40 ℃ | ns | ** | ns | *** | 0.0670 | 0.0015 | 0.0974 | 0.0007 |
| 10^-4^ M | 28 ℃ vs. 34 ℃ | ** | ** | * | ** | 0.0050 | 0.0022 | 0.0261 | 0.0046 |
|  | 28 ℃ vs. 40 ℃ | ** | **** | ns | ns | 0.0040 | <0.0001 | 0.3173 | 0.6241 |
|  | 34 ℃ vs. 40 ℃ | ns | *** | ns | ** | 0.7578 | 0.0004 | 0.0748 | 0.0063 |
| 10^-3^ M | 28 ℃ vs. 34 ℃ | * | * | * | *** | 0.0384 | 0.0303 | 0.0417 | 0.0007 |
|  | 28 ℃ vs. 40 ℃ | ns | *** | ns | ns | 0.0781 | 0.0002 | 0.4261 | 0.0706 |
|  | 34 ℃ vs. 40 ℃ | ** | *** | ns | ** | 0.0080 | 0.0005 | 0.1100 | 0.0013 |

*indicates *p*<0.05; **indicates *p*<0.01; ***indicates *p*<0.001; ****indicates *p*<0.0001; ns indicates not significant.

**Table S49.** Two-way ANOVA results for the effects of temperature and putrescine concentration on ASA.

| Factors | Day 2 | | Day 4 | | Day 6 | | Day 8 | |
| --- | --- | --- | --- | --- | --- | --- | --- | --- |
|  | F | *p* | F | *p* | F | *p* | F | *p* |
| Temperature | 5.917 | <0.05 | 55.695 | <0.001 | 22.934 | <0.001 | 141.289 | <0.001 |
| Putrescine | 17.486 | <0.001 | 9.480 | <0.001 | 5.158 | <0.05 | 10.564 | <0.001 |
| Temperature* Putrescine | 2.127 | 0.079 | 7.759 | <0.001 | 2.564 | <0.05 | 5.881 | <0.001 |

**Table S50.** Post-hoc comparisons of the temperature effect on ASA within each putrescine concentration.

| Dunnett's multiple comparisons test | | Summary | | | | Adjusted *p* value | | | |
| --- | --- | --- | --- | --- | --- | --- | --- | --- | --- |
|  |  | Day 2 | Day 4 | Day 6 | Day 8 | Day 2 | Day 4 | Day 6 | Day 8 |
| 0 M | 28 ℃ vs. 34 ℃ | ns | * | * | ** | >0.9999 | 0.0136 | 0.0147 | 0.0017 |
|  | 28 ℃ vs. 40 ℃ | ns | * | *** | ns | 0.1948 | 0.0438 | 0.0009 | 0.0908 |
|  | 34 ℃ vs. 40 ℃ | ns | ** | *** | *** | 0.1944 | 0.0032 | 0.0004 | 0.0009 |
| 10^-7^ M | 28 ℃ vs. 34 ℃ | ns | ns | ns | * | >0.9999 | 0.3760 | 0.5872 | 0.0297 |
|  | 28 ℃ vs. 40 ℃ | ns | *** | ns | ns | 0.5773 | 0.0002 | 0.0654 | 0.3909 |
|  | 34 ℃ vs. 40 ℃ | ns | *** | * | * | 0.5773 | 0.0002 | 0.0340 | 0.0143 |
| 10^-6^ M | 28 ℃ vs. 34 ℃ | ns | * | ns | ns | >0.9999 | 0.0168 | 0.9482 | 0.0534 |
|  | 28 ℃ vs. 40 ℃ | ** | *** | ns | ns | 0.0013 | 0.0007 | 0.4553 | 0.9719 |
|  | 34 ℃ vs. 40 ℃ | ** | *** | ns | * | 0.0013 | 0.0003 | 0.5966 | 0.0463 |
| 10^-5^ M | 28 ℃ vs. 34 ℃ | ns | ns | ** | ns | >0.9999 | 0.9631 | 0.0032 | 0.0531 |
|  | 28 ℃ vs. 40 ℃ | ns | ns | * | ns | 0.7966 | 0.9231 | 0.0337 | 0.2732 |
|  | 34 ℃ vs. 40 ℃ | ns | ns | ** | * | 0.7952 | 0.8060 | 0.0011 | 0.0187 |
| 10^-4^ M | 28 ℃ vs. 34 ℃ | ns | ns | ns | ** | >0.9999 | 0.9775 | 0.1589 | 0.0027 |
|  | 28 ℃ vs. 40 ℃ | * | * | * | * | 0.0242 | 0.0175 | 0.0280 | 0.0320 |
|  | 34 ℃ vs. 40 ℃ | * | * | ** | ** | 0.0242 | 0.0192 | 0.0091 | 0.0010 |
| 10^-3^ M | 28 ℃ vs. 34 ℃ | ns | ns | ns | ns | >0.9999 | 0.9416 | 0.2191 | 0.3735 |
|  | 28 ℃ vs. 40 ℃ | ** | ns | * | ns | 0.0071 | 0.9644 | 0.0349 | 0.7356 |
|  | 34 ℃ vs. 40 ℃ | ** | ns | ns | ns | 0.0071 | 0.9968 | 0.1547 | 0.1861 |

*indicates *p*<0.05; **indicates *p*<0.01; ***indicates *p*<0.001; ****indicates *p*<0.0001; ns indicates not significant.

**Table S51.** Two-way ANOVA results for the effects of temperature and putrescine concentration on MDA.

| Factors | Day 2 | | Day 4 | | Day 6 | | Day 8 | |
| --- | --- | --- | --- | --- | --- | --- | --- | --- |
|  | F | *p* | F | *p* | F | *p* | F | *p* |
| Temperature | 11.506 | <0.001 | 67.173 | <0.001 | 29.634 | <0.001 | 469.18 | <0.001 |
| Putrescine | 0.698 | 0.092 | 8.557 | <0.001 | 3.732 | <0.05 | 23.24 | <0.001 |
| Temperature* Putrescine | 1.616 | 0.092 | 18.519 | <0.001 | 3.521 | <0.001 | 31.225 | <0.001 |

**Table S52.** Post-hoc comparisons of the temperature effect on MDA within each putrescine concentration.

| Dunnett's multiple comparisons test | | Summary | | | | Adjusted *p* value | | | |
| --- | --- | --- | --- | --- | --- | --- | --- | --- | --- |
|  |  | Day 2 | Day 4 | Day 6 | Day 8 | Day 2 | Day 4 | Day 6 | Day 8 |
| 0 M | 28 ℃ vs. 34 ℃ | ns | *** | ns | ** | 0.2919 | 0.0004 | 0.0558 | 0.0055 |
|  | 28 ℃ vs. 40 ℃ | ** | ** | ns | ns | 0.0077 | 0.0013 | 0.0567 | 0.6748 |
|  | 34 ℃ vs. 40 ℃ | * | * | ns | ** | 0.0159 | 0.0126 | 0.9996 | 0.0074 |
| 10^-7^ M | 28 ℃ vs. 34 ℃ | ns | * | ns | ** | 0.3354 | 0.0361 | 0.8340 | 0.0012 |
|  | 28 ℃ vs. 40 ℃ | ns | ns | ns | ns | 0.8895 | 0.5324 | 0.5454 | 0.0677 |
|  | 34 ℃ vs. 40 ℃ | ns | ns | ns | ** | 0.5110 | 0.0763 | 0.8427 | 0.0025 |
| 10^-6^ M | 28 ℃ vs. 34 ℃ | ns | ** | ns | *** | 0.6073 | 0.0078 | 0.0757 | 0.0007 |
|  | 28 ℃ vs. 40 ℃ | ns | ns | ns | ** | 0.9992 | 0.8384 | 0.0839 | 0.0073 |
|  | 34 ℃ vs. 40 ℃ | ns | ** | ns | ** | 0.5888 | 0.0064 | 0.9885 | 0.0034 |
| 10^-5^ M | 28 ℃ vs. 34 ℃ | ns | ns | * | *** | 0.3882 | 0.4051 | 0.0234 | 0.0001 |
|  | 28 ℃ vs. 40 ℃ | ns | ns | ns | ns | 0.5259 | 0.0732 | 0.9735 | 0.9997 |
|  | 34 ℃ vs. 40 ℃ | ns | * | * | *** | 0.9389 | 0.0295 | 0.0261 | 0.0001 |
| 10^-4^ M | 28 ℃ vs. 34 ℃ | ns | ns | * | ** | 0.1937 | 0.4357 | 0.0125 | 0.0017 |
|  | 28 ℃ vs. 40 ℃ | ns | ns | * | ns | 0.8879 | 0.5167 | 0.0359 | 0.3986 |
|  | 34 ℃ vs. 40 ℃ | ns | ns | ns | ** | 0.1311 | 0.1486 | 0.2161 | 0.0025 |
| 10^-3^ M | 28 ℃ vs. 34 ℃ | ns | ns | ns | ** | 0.1320 | 0.4638 | 0.3126 | 0.0046 |
|  | 28 ℃ vs. 40 ℃ | ns | * | * | * | 0.0898 | 0.0143 | 0.0214 | 0.0107 |
|  | 34 ℃ vs. 40 ℃ | * | * | ns | ns | 0.0180 | 0.0269 | 0.0582 | 0.1633 |

*indicates *p*<0.05; **indicates *p*<0.01; ***indicates *p*<0.001; ****indicates *p*<0.0001; ns indicates not significant.

**Table S53.** Two-way ANOVA results for the effects of temperature and putrescine concentration on NO.

| Factors | Day 2 | | Day 4 | | Day 6 | | Day 8 | |
| --- | --- | --- | --- | --- | --- | --- | --- | --- |
|  | F | *p* | F | *p* | F | *p* | F | *p* |
| Temperature | 19.701 | <0.001 | 331.444 | <0.001 | 21.577 | <0.001 | 11.300 | <0.001 |
| Putrescine | 0.891 | 0.092 | 7.277 | <0.001 | 19.559 | <0.001 | 14.050 | <0.001 |
| Temperature* Putrescine | 1.268 | 0.092 | 11.229 | <0.001 | 7.927 | <0.001 | 3.361 | <0.05 |

Table S54. Post-hoc comparisons of the temperature effect on NO within each putrescine concentration.

| Dunnett's multiple comparisons test | | Summary | | | | Adjusted *p* value | | | |
| --- | --- | --- | --- | --- | --- | --- | --- | --- | --- |
|  |  | Day 2 | Day 4 | Day 6 | Day 8 | Day 2 | Day 4 | Day 6 | Day 8 |
| 0 M | 28 ℃ vs. 34 ℃ | * | ns | ** | ns | 0.0175 | 0.2689 | 0.0021 | 0.0755 |
|  | 28 ℃ vs. 40 ℃ | * | ns | *** | ns | 0.0149 | 0.1698 | 0.0001 | 0.0693 |
|  | 34 ℃ vs. 40 ℃ | ns | ns | ns | ns | 0.9937 | 0.9440 | 0.0796 | 0.9983 |
| 10^-7^ M | 28 ℃ vs. 34 ℃ | ns | ns | ns | *** | 0.0806 | 0.2489 | 0.5850 | 0.0003 |
|  | 28 ℃ vs. 40 ℃ | ns | ns | ** | *** | 0.1344 | 0.5246 | 0.0023 | 0.0006 |
|  | 34 ℃ vs. 40 ℃ | ns | ns | ** | ns | 0.9403 | 0.8233 | 0.0098 | 0.8890 |
| 10^-6^ M | 28 ℃ vs. 34 ℃ | ns | * | ns | ns | 0.4905 | 0.0248 | 0.1778 | 0.6219 |
|  | 28 ℃ vs. 40 ℃ | ns | ns | ns | ns | 0.6633 | 0.5521 | 0.3567 | 0.6515 |
|  | 34 ℃ vs. 40 ℃ | ns | ns | ns | ns | 0.9515 | 0.1307 | 0.8694 | 0.2061 |
| 10^-5^ M | 28 ℃ vs. 34 ℃ | ns | ns | ns | ns | 0.2393 | 0.1373 | 0.1062 | 0.1901 |
|  | 28 ℃ vs. 40 ℃ | ns | ns | ns | ns | 0.2978 | 0.7741 | 0.8784 | 0.2392 |
|  | 34 ℃ vs. 40 ℃ | ns | ns | * | ns | 0.9859 | 0.3607 | 0.0499 | 0.9857 |
| 10^-4^ M | 28 ℃ vs. 34 ℃ | ns | * | ns | ns | 0.2465 | 0.0121 | 0.1632 | 0.5295 |
|  | 28 ℃ vs. 40 ℃ | ns | ns | ns | ns | 0.6541 | 0.3128 | 0.7013 | 0.4916 |
|  | 34 ℃ vs. 40 ℃ | ns | ns | ns | ns | 0.6941 | 0.1327 | 0.4823 | 0.1075 |
| 10^-3^ M | 28 ℃ vs. 34 ℃ | ** | ns | **** | ns | 0.0095 | 0.1246 | <0.0001 | 0.5461 |
|  | 28 ℃ vs. 40 ℃ | * | ns | **** | * | 0.0232 | 0.5124 | <0.0001 | 0.0312 |
|  | 34 ℃ vs. 40 ℃ | ns | ns | ns | ns | 0.8311 | 0.5633 | 0.4877 | 0.1654 |

*indicates *p*<0.05; **indicates *p*<0.01; ***indicates *p*<0.001; ****indicates *p*<0.0001; ns indicates not significant.
